# The Anthranil Core as a π‑Conjugated Bridge in the Synthesis of Molecular Photosensitizers

**DOI:** 10.1021/acs.joc.5c00389

**Published:** 2025-07-17

**Authors:** Laia Marín Moncusí, Carlos E. Puerto Galvis, Eugenia Martínez-Ferrero, Emilio Palomares

**Affiliations:** † Institute of Chemical Research of Catalonia-The Barcelona Institute of Science and Technology (ICIQ-BIST), Avda. Països Catalans 16, 43007 Tarragona, Spain; ‡ Departament d’Enginyeria Electrònica, Elèctrica i Automàtica. Universitat Rovira i Virgili, Avda. Països Catalans 26, 43007 Tarragona, Spain; § Catalan Institution for Research and Advanced Studies (ICREA), Passeig Lluís Companys 23, 08010 Barcelona, Spain

## Abstract

Molecular photosensitizers based on the anthranil core were synthesized through a six-step linear synthesis featuring a Suzuki coupling at the C7 position and a controlled C3–H arylation. The introduction of donor and acceptor groups allowed the synthesis of unsymmetrical photosensitizers that were then investigated, revealing different spectroscopic and optoelectronic properties. Transient spectroscopy of excited species indicated that placing the acceptor at C7 and the donor group at C3 altered the energy levels around the anthranil core, making these dyes attractive for photovoltaic applications.

## Graphical abstract

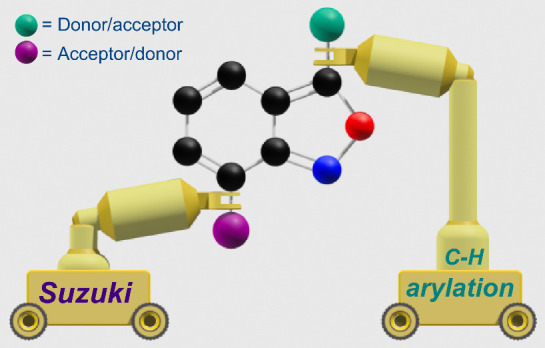

With the energy demand rising every day, our society needs to focus on pursuing strategies to move away from fossil-based systems and promote low-carbon and renewable resources to produce energy, such as wind and solar.^1^ One of the most promising approaches to indoor powering utilities for converting light into electricity is dye-sensitized solar cells (DSSCs), which have reached a power conversion efficiency (PCE) of 15.2% thanks to optimized molecular photosensitizers.^2^ The three leading families of photosensitizers for DSSCs include Ru­(II) polypyridyl complexes, Zn porphyrin derivatives, and metal-free organic dyes. Through careful design and synthesis, the properties of the organic photosensitizers can be tuned to obtain a high absorption coefficient and a broad spectral response to enhance the photovoltaic performance.^3^

The chemical architecture of an organic photosensitizer involves an electron donor (D) group linked to an electron acceptor (A) moiety through a π-conjugated bridge (π).^4^ To date, the most popular π-conjugated linkers are chromophores, such as BODIPY, isoindigo, and porphyrins^5^ and *N*- and/or *S*-heterocycles, such as benzothiadiazole, quinoxaline, diketopyrrolopyrrole, and benzotriazole.^6^ Among them, electron-deficient benzo­[*c*]­[2,1,3]­thiadiazole **1** is one of the most widely studied units for DSSCs due to its capability to tune the energy bandgap to maximize the light absorption, allowing for record PCEs in cosensitized DSSCs.^7,8^ Almost all of the benzothiadiazole-based molecules for DSSCs present a typical substitution pattern where the donor/acceptor (D/A) groups are at the 4 and 7 positions. Under this approach, Zhang et al. recently reported the synthesis of a simple D-π-A dye **2**, the MS5 compound commercialized by Dyenamo, that enabled the fabrication of a cosensitized DSSC with a PCE of 13.5% and a *V*_oc_ of 1.24 V (Figure 1).^9^

**Figure 1:**
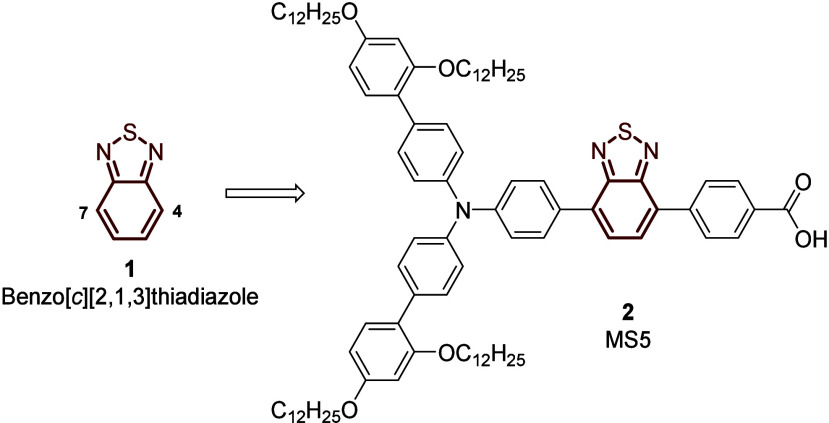
Structures of benzo­[*c*]­[2,1,3]­thiadiazole **1** and commercial dye MS5 **2**.

The benzothiadiazole structure combines a five-membered ring containing N and S atoms and a benzene ring. Both rings possess different electron densities; however, the donor/acceptor groups can only be placed at the benzene ring, leading to a symmetrical substitution pattern at positions 4 and 7 such as in MS5.

After scouting different heterocycles, we identified a chemical entity that could be used as π-conjugated bridge in the synthesis of new photosensitizers, having a five-membered ring heterocycle that has a C–H bond available for further substitutions and is fused with a benzene ring.

Thus, we envisioned that the anthranil core **3** would be suitable to place one group (donor/acceptor) at the five-membered ring and the other group at the benzene ring (Figure 2), preparing novel molecular photosensitizers with different charge transfer properties, e.g., by placing the donor group at the electron rich ring and the acceptor group at the electron poor ring, or vice versa.

**Figure 2:**
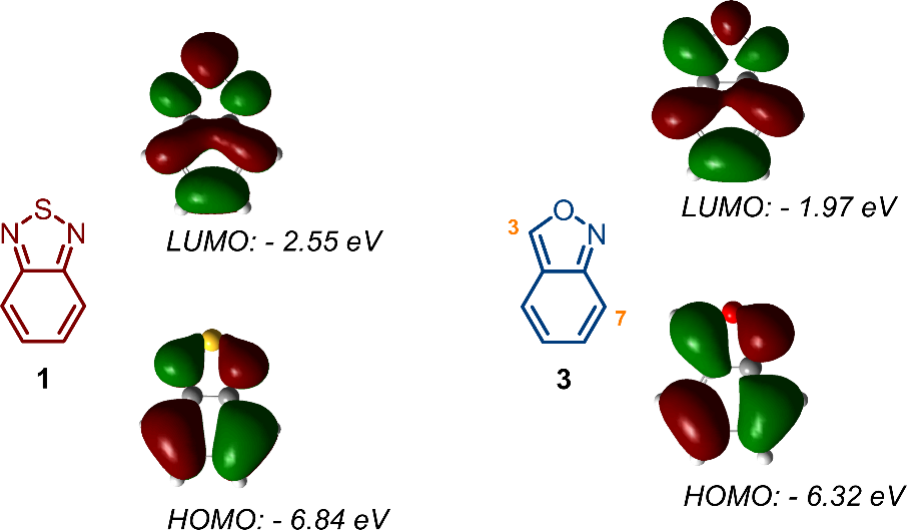
Frontier molecular orbitals for **1** and **3** according to density functional theory (DFT).

Anthranils (2,1-benzisoxazoles) were discovered in the 1960s, and they have been privileged scaffolds for medicinal chemistry and versatile building blocks for the synthesis of more complex structures.^10,11^ However, to the best of our knowledge, they have not been used in materials science or in DSSCs. Compared with **1**, the anthranil core **3** possesses similar HOMO–LUMO energy levels and a moderate electron density at the isoxazole ring that ensures its use as a π-conjugated bridge (Figure 2). Moreover, from a synthetic point of view, the desired substitution in this skeleton involves the challenging formation of two C–C bonds, which motivated us to consider potential synthetic routes to functionalize the C3 and C7 positions at the anthranil core.

Our synthetic approach started with the synthesis of the corresponding bromo-substituted anthranil **5** (78%) through a reductive cyclization of commercial *o*-nitrobenzaldehyde **4** using SnCl_2_ as a reductive agent under smooth reaction conditions (Scheme 1).^12^ Then, the reactive site at C7 in derivative **5** allowed us to select the Suzuki–Miyaura coupling as the second step in our synthetic plan over the C–H arylation reaction. In this order, we first introduce the corresponding donor/acceptor moiety at the C7 position using the synthetic boronic ester **6** to insert the triphenylamine core as a donor group and give the intermediate **7** in 85% yield, while with the commercial boronic acid **8** the intermediate with the benzoic acid–methyl ester moiety as the acceptor group was obtained in 75% yield (Scheme 1).^13^

**Scheme 1:**
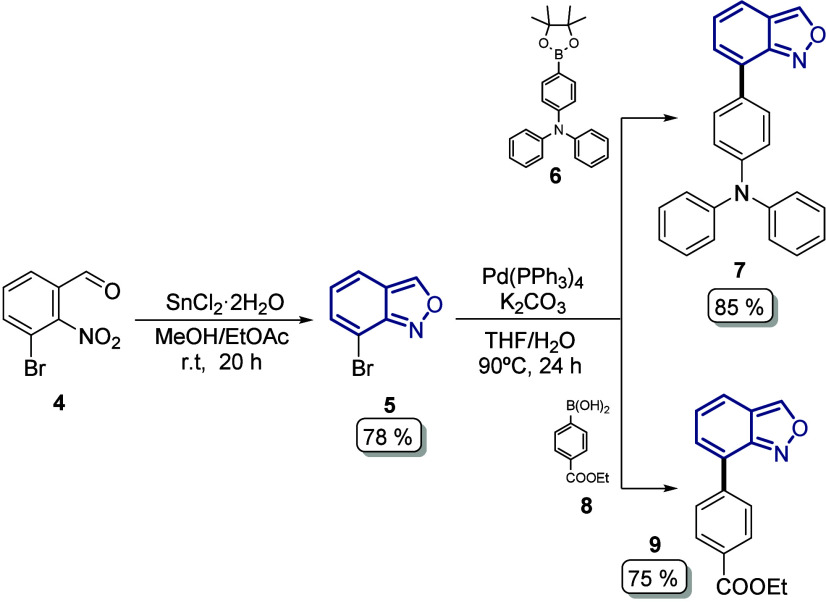
Preparation of Anthranil Derivatives with the Donor (**7**) and Acceptor (**9**) Moieties at the C7 Position

Considering the difficulty of accessing aryl diazonium tetrafluoroborates^14^ and the regioselectivity issues^15^ of the recently reported metal-free C–H arylation of anthranils, we decided to explore a Pd catalytic system, optimizing the reaction conditions for the selective C3-arylation on compound **5** by first using the commercial anthranil **5′** and 4-bromoanisole **10′** as model substrates (Table 1). We successfully obtained our desired product **11′** in 88% yield using Pd­(OAc)_2_ as a catalyst and KOAc as the base and performing the reaction at 150 °C for 24 h in DMA as a solvent (entry 8, Table 1).

**Table 1:**
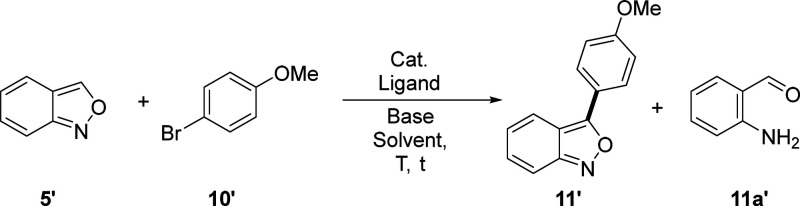
Optimization of Reaction Conditions for the C–H Arylation Using Model Substrates **5′** and **10′**

Entry	Cat. (mol %)	Ligand (mol %)	Base (2 equiv)	Solvent (5 mL)	*T* (°C)	Time (h)	Yield (%)​^b^
1	Pd_2_dba_3_ (1)		K_2_CO_3_	DMA	150	24	NR​^c^
2	Pd_2_dba_3_ (1)		Cs_2_CO_3_	DMA	150	24	NR​^c^
3	PdCl_2_ (1)	PPh_3_ (1.5)	KOAc	DMA	150	24	40​
4	Pd(PPh_3_)_4_ (1)		KOAc	DMA	150	24	21​
5	Pd_2_dba_3_ (1)		NaO*t*Bu	DMA	150	24	45​
6	Pd_2_dba_3_ (1)		KO*t*Bu	DMA	150	24	40​
7	Pd(OAc)_2_ (1)	P(*t*-Bu)_3_ (1.5)	KOAc	DMA	150	24	32​
8	Pd(OAc)_2_ (1)		KOAc	DMA	150	24	88​
9	Pd(OAc)_2_ (1)		KOAc	DMF	150	24	44​
10	Pd(OAc)_2_ (1)		KOAc	DMA	120	24	30​
11	Pd(OAc)_2_ (1)		KOAc	DMA	150	12	40​
12	Pd(OAc)_2_ (10)		KOAc	DMA	150	24	51​^d^

a Reaction conditions on a 1 mmol scale: anthranil **5′** (1.5 equiv), bromoanisole **10′** (1 equiv), base (2 equiv), solvent (5 mL), temperature, and time.

b Isolated yields.

c NR: No Reaction.

d **11a′** was identified by GC-MS upon increasing the catalyst loading.

Subsequent modifications to the standard conditions revealed that palladium catalysts such as Pd_2_dba_3_, PdCl_2_, and Pd­(PPh_3_)_4_, combined with common phosphine ligands like PPh_3_ and P­(*t*-Bu)_3_, did not lead to yields of the desired product over 40%. Furthermore, the use of bases such as K_2_CO_3_, Cs_2_CO_3_, NaOtBu, and KOtBu to promote the abstraction of the C–H bond at C3 was ineffective in comparison to KOAc.

Regarding the solvent, DMA was chosen in the first place as an aprotic and high-boiling-point reaction media to perform the experiments described in Table 1. Attempts to change to DMF decreased the yield of **11′** to 44% under the same conditions: catalyst, base and temperature (entry 9, Table 1).

A decrease in the reaction temperature (120 °C) or reaction time (12 h) dramatically decreased the reaction yields compared to the standard conditions. Finally, increasing the catalyst loading (10 mol %) favored the *in situ* ring opening of the starting anthranil, forming byproducts derived from nitrene and ketene intermediates.^16^

With the optimized reaction conditions, we applied this protocol to couple the donor substrate **7** with the commercial ethyl 4-bromobenzoate **10**, obtaining the D-anthranil-A intermediate **11** in an excellent yield (88%) due to the enriched electron density over the anthranil core given by the attached triphenylamine group present at the C7 position of substrate **7** (Scheme 2).

**Scheme 2:**
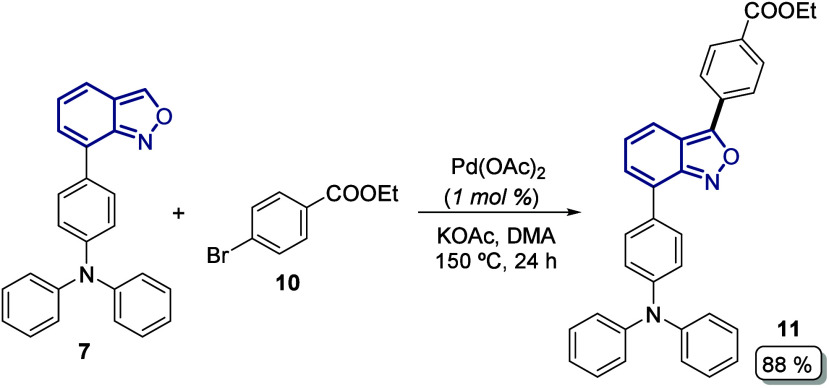
C3-Arylation on the Donor Anthranil Core **7** to give the D-Anthranil-A intermediate **11**

On the other hand, when the same protocol for the C3 arylation was applied to substrate **9** with the commercial 4-bromo-*N*,*N*-diphenylaniline **12**, which resulted in a moderate yield of desired A-anthranil-D intermediate **13** (70%) due to the formation of a byproduct **13′** in considerable amounts (27%). In this case, the formation of the side product was promoted by the presence of the acceptor benzoate moiety at the C7 position of anthranil **9**, reducing the reactivity of the C3–H bond and favoring the ring opening of the five-membered ring of the anthranil to give the corresponding aldehyde **13′** (Scheme 3).

**Scheme 3:**
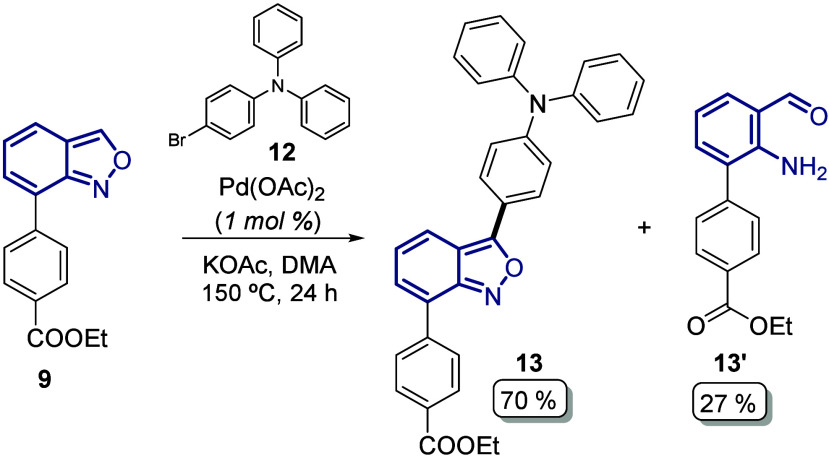
C3-Arylation on the Aceptor Anthranil Core **9** to give the D–Anthranil–A Intermediate **13** and the Byproduct **13′**

The final steps of our synthetic approach for the synthesis of the novel anthranil-based photosensitizers were directed toward the construction of the bulky donor moiety: the *N*-(2′,4′-bis­(dodecyloxy)-[1,1′-biphenyl]-4-yl)-2′,4′-bis­(dodecyloxy)-*N*-phenyl-[1,1′-biphenyl]-4-amine fragment, also known as the Hagfeldt donor. Thus, intermediates **11** and **13** were subjected to a selective aromatic dibromination using NBS in DMF to afford compounds **14** (73%) and **18** (93%) in good to excellent yields (Scheme 4).

**Scheme 4:**
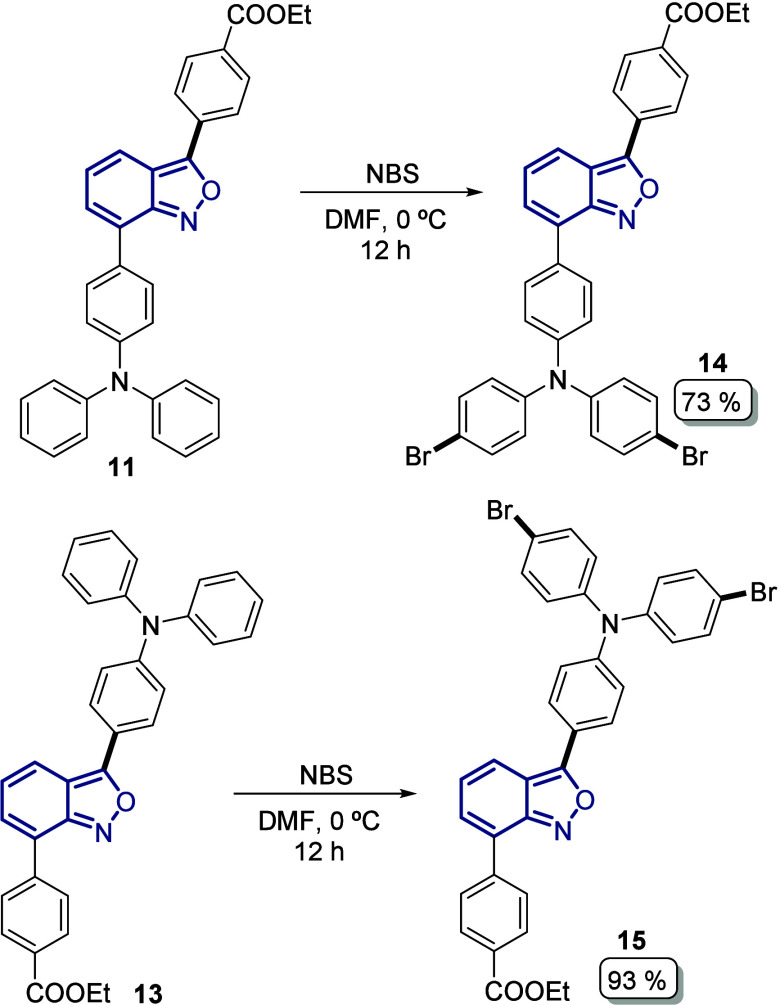
Di-Bromination at the Diphenyl Moiety in Anthranil Derivatives **11** and **13**

Compounds **14** and **15** were used then as the starting material for the Suzuki–Miyaura coupling with the synthetic boronate ester **16** to introduce the 2,4-bis­(dodecyloxy)­benzene moiety, furnishing the unsymmetric anthranil benzoates **17** (72%) and **18** (81%) in good yields bearing the donor and acceptor groups at the C3 and C7 positions (Scheme 5a). Finally, hydrolysis was performed under basic and mild conditions to give the desired photosensitizers LM14 **19** and CP104 **20** with excellent yields of 90% and 97%, respectively (Scheme 5a). Our target compounds were obtained on a 300 mg scale in a linear seven-step synthetic route and with satisfactory overall yields of 27% and 29%, respectively, for **19** and **20**.

**Scheme 5:**
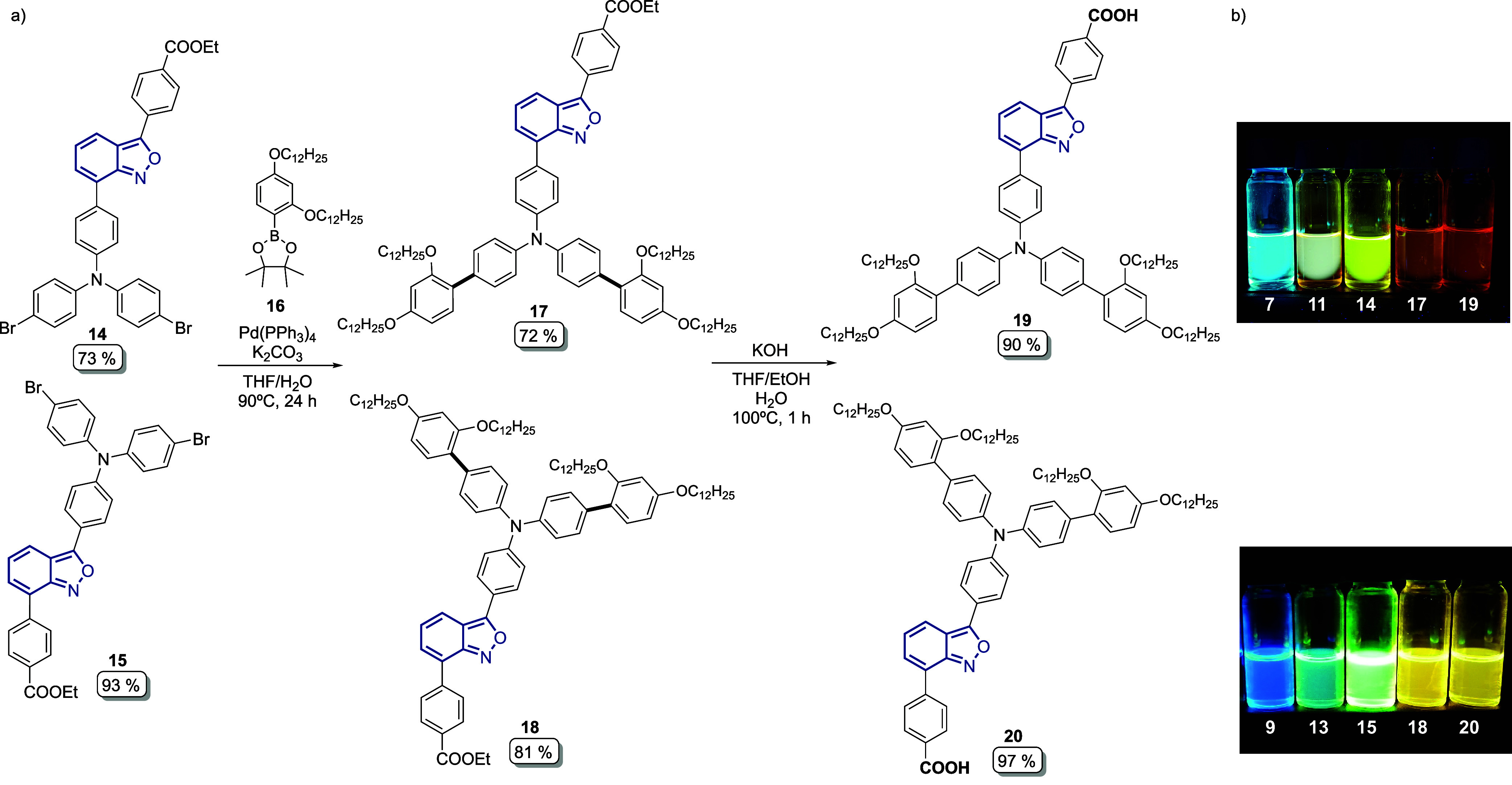
^a^

a (a) Construction of the Hagfeldt donor fragment in the anthranil core to complete the synthesis of photosensitizers **19** and **20**. (b) Change in the light emission during the chemical transformation of intermediates **7** and **9** into photosensitizers **19** and **20**, respectively (THF at 365 nm).

In the synthesis of **19** and **20**, we noticed that during the introduction of the donor and acceptor groups into the structure of anthranil **5**, the resulting intermediates started to exhibit interesting photoluminescence properties due to having different groups attached to various positions of the anthranil core (Scheme 5b). In the case of photosensitizer **19** and its intermediates, we observed that by having the donor group at the C7 position and the acceptor group at C3, the emission of **7** moved from 497 nm (blue) to 643 nm (red) through all the chemical transformations to access the final compound **19** (Figure S1). This effect can be explained by the fact that in **19**, the acceptor group at C3 is directly attached to the more electron-dense ring of the anthranil core, favoring the movement of charges from the donor to the acceptor moiety and resulting in a lower energy gap between the HOMO and LUMO of **19** in comparison with those of **20**. On the other side, having the donor group at the C3 position of **20** completely changes the distribution of the electron density in the molecule through the anthranil bridge, affecting the movement of charges and increasing the energy bandgap between the HOMO and LUMO of **20**, shifting the emission to 577 nm (yellow) (Scheme 5b).

We also studied and characterized our target compounds to evaluate their potential as photosensitizers in photovoltaic applications (Figure S2). These studies were complemented by cyclic voltammetry (CV) measurements of the three dyes in solution using a three-electrode electrochemical cell to depict their energy level alignments (Figure S3). With these data (UV–vis and CV experiments), we calculated the optical bandgap (*E*_g_) and molecular energy levels for compounds **19** and **20** to estimate their ability to promote and inject electrons in photovoltaic devices. These values are summarized in Table S1.

To shed light on the charge transfer capabilities between the dyes and the semiconductors such as TiO_2_, we first performed time-correlated single photon counting (TCSPC) of the dyes in solution (Figure S4 and Table S2), and then we recorded the transient absorption spectra (TAS) to elucidate the charge transfer kinetics of dyes **2**, **19**, and **20** sensitized on 4 μm TiO_2_ films (Figure S5).

The regeneration kinetics of the excited states of the dyes **2**, **19**, and **20** were studied at 920 nm in films with and without electrolyte ([Cu^(II/I)^(tmby)_2_]­[TFSI]_2/1_) (Figures S6 and S7).

As was expected, in the absence of an electrolyte, recombination of the photoinjected electrons in TiO_2_ and the oxidized dye is slower. However, the faster regeneration in the presence of the electrolyte can be explained by the energy of the HOMO level, which promotes a higher driving force for the electrolyte regeneration (Figures S8).^17^

In conclusion, we have designed and developed an efficient synthetic route, based on Suzuki coupling and C–H arylation, to introduce the anthranil scaffold as a π-conjugated bridge in the structures of novel molecular photosensitizers. The selective C–H activation was studied using different Pd catalysts, establishing a protocol for the controlled C3 arylation of anthranils substituted with donor or acceptor groups in good yields and preventing side reactions and the ring opening of the anthranilic core. We found that placing donor and acceptor groups at the C3 and C7 positions ultimately changes the electron density within the molecule, altering their energetic levels and the nature of the excited species and resulting in compound **20** being more efficient for electron regeneration on solid-state films. Overall, we report the synthesis of two new anthranil-based photosensitizers with fast excited state lifetimes and properties that make them candidates for the fabrication of cosensitized DSSCs and low-intensity illumination devices.

## Supplementary Material





## Data Availability

The study’s data are available in the published article and its Supporting Information.

## Notes

Detailed experimental procedures, NMR spectra, and photophysics data (PDF)
FAIR data, including the primary NMR FID files, for compounds **5**–**20** (ZIP)
