# Supplementary material for: The Anthranil Core as a π‑Conjugated Bridge in the Synthesis of Molecular Photosensitizers
Source: J Org Chem. 2025 Jul 17;90(30):10908–12. doi: 10.1021/acs.joc.5c00389 (PMC12418303; doi:10.1021/acs.joc.5c00389)
Supplement: Supplementary file 1 [file jo5c00389_si_001.pdf]

The anthranil core as π-conjugated bridge in the synthesis of molecular photosensitizers

Laia Marín Moncusí,[a,b] Carlos E. Puerto Galvis,[*a] Eugenia Martínez-Ferrero[a] and Emilio Palomares[*ac]

[a]*Institute of Chemical Research of Catalonia-The Barcelona Institute of Science and Technology (ICIQ-BIST), Avda. Països Catalans, 16, Tarragona, Spain. E-mail: cpuerto@iciq.es, epalomares@iciq.es*
[b]*Departament d'Enginyeria Electrònica, Elèctrica i Automàtica. Universitat Rovira i Virgili, Avda. Països Catalans, 26, Tarragona, Spain.*
[c]*Catalan Institution for Research and Advanced Studies (ICREA), Passeig Lluís Companys 23, Barcelona, Spain.*

ELECTRONIC SUPPORTING INFORMATION

List of contents

1. **General information** **SI 2**
2. **Experimental details and characterization data of starting materials** **SI 3**
3. **Characterization data for the side product 13'** **SI 5**
4. **Experimental details and characterization data of photosensitiser 19** **SI 5**
5. **Experimental details and characterization data of photosensitiser 20** **SI 8**
6. **Absorption and emission spectra of selected photosensitizers 19** **SI 12**
7. **UV–Vis and fluorescence spectra for dyes MS5 2, 19 and 20** **SI 12**
8. **Cyclic voltammogram curves of dyes MS5, 19 and 20.** **SI 12**
9. **Optical and electrochemical properties of dyes MS5, 19 and 20.** **SI 13**
10. **Time-Correlated Single Photon Counting of photosensitizers in solution** **SI 13**
11. **TAS measurements on 4 µm transparent TiO_2 sensitized films** **SI 14**
12. **Transient Absorption Spectra (TAS) experiments** **SI 15**
13. **Transient absorption decays for dyes MS5, 19 and 20** **SI 15**
14. **Copies of the ^{1}H and ^{13}C NMR Spectra** **SI 16**

1. General information

Unless otherwise specified, all chemicals, reagents, and solvents used in the synthesis of compounds **5-20** were obtained from commercial sources in reagent-grade quality and were employed without additional purification. Sensitive reactions were conducted under an argon atmosphere using standard Schlenk techniques (under high-vacuum, ~10^{-2} mbar) with solvents that were distilled and dried, unless otherwise noted. The progress of the reactions was monitored by thin-layer chromatography (TLC) using Merck silica gel 60 F254 precoated plates (0.25 mm) and visualized with UV lamp. All workup and purification procedures were carried out using reagents and solvents of analytical grade. Flash column chromatography was performed on silica gel (Aldrich, 230-400 mesh) using hexane, dichloromethane and THF (99.8 % stabilized with BHT) as eluents. Organic solutions were concentrated under reduced pressure on a Büchi rotatory evaporator. Unless otherwise stated, reactions were carried out under argon atmosphere. Yields refer to purified compounds unless otherwise noted. ^{1}H and ^{13}C NMR spectra were recorded at 298 K on Bruker Avance 400 Ultrashield or Bruker Avance 500 Ultrashield apparatuses. Coupling constants (*J*) are quoted in hertz (Hz). Multiplicity is reported with the following abbreviations: s = singlet, brs = broad singlet, d = doublet, t = triplet, q = quartet, dt = doublet of triplets, td = triplet of doublets, tt = triplet of triplets, sp = septet, m = multiplet, app = apparent. Melting points were measured using open glass capillaries in a Büchi B540 apparatus. Infrared spectra were recorded on a Bruker Tensor 27. Mass spectra were recorded on a Waters LCT Premier spectrometer. Gas chromatography-mass spectrometry (GC-MS) was carried out in Agilent 7890B - 5877A MSD. Transient absorption measurements were carried on by employing a NdYAGlaser with OPO tuned at 420 nm, 10 ns pulse width and 600uJ of power. A halogen-tungsten lamp with double monochromator formed the light probe, and the optical signal was detected by an amplified silicon photodetector coupled to a datalogger. Photoluminescent lifetime measurements were carried out on a Edinburgh Instruments LifeSpec-II based on the time-correlated single photon counting (TCSPC) technique, equipped with a detector, double subtractive monochromator and picosecond pulsed diode lasers source. The decay kinetics were fitted using a biexponential decay model: $y = A_1 \exp(-t/\tau_1) + A_2 \exp(-t/\tau_2)$, where τ_1 and τ_2, represent the lifetime decay. Current density-voltage (J-V) measurements were conducted under standard conditions, which involved an intensity equivalent to 1 sun (100 mW cm^{-2}, AM 1.5G, 25 °C). These measurements were carried out utilizing a Keithley 2400 source meter and an ABET Technologies solar simulator (model 11000, class type A).

2. Experimental details and characterization data of starting materials

- ***hesis of 7-bromobenzo[c]isoxazole 5:***

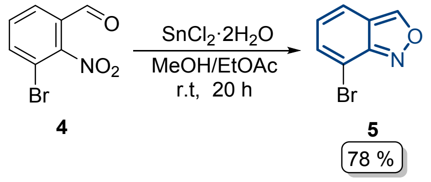

In a 250 mL round-bottom flask a mixture of 3-bromo-2-nitrobenzaldehyde **4** 1.52 g (6.60 mmol) and $SnCl_2·2H_2O$ 4.48 g (19.8 mmol, 3 equiv) were dissolved in 60 mL of MeOH and 30 mL of EtOAc and the solution was left stirring at room temperature for 20 hours. Upon completion of the reaction, the solvents were evaporated by vacuum and the reaction mixture was neutralized with a saturated aqueous $NaHCO_3$ solution (1 M). Next, the mixture was extracted with DCM and dried over anhydrous magnesium sulphate. The solvent was evaporated and the crude product was purified by a silica gel column chromatography using *n*-hexane/THF (4:1) as eluent. After purification, 0.51 g (2.58 mmol, 78% yield) of the desired product **5** were obtained as a dark orange oil. R_f =0.34 (*n*-hexane/THF = 4:1 v/v). ^{1}H NMR (500 MHz, $CDCl_3$) $\delta_{(ppm)}$: 9.26 (s, 1H), 7.57-7.51 (m, 2H), 6.90 (dd, *J* = 8.7, 6.9 Hz, 1H). ^{13}C {^{1}H} NMR (126 MHz, $CDCl_3$) $\delta_{(ppm)}$: 156.3, 155.7, 133.8, 125.3, 119.2, 119.1, 108.9. HRMS (ESI) m/z calcd. for (C_7H_5BrNO) $[M+H]^+$ 197.9549, found 197.9546.

- ***Synthesis of N,N-diphenyl-4-(4,4,5,5-tetramethyl-1,3,2-dioxoborolan-2-yl)aniline 6:***

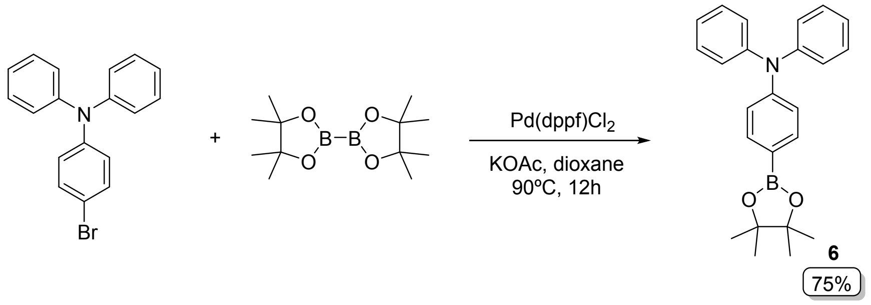

In 250 mL round-bottom flask 1.81 g (5.60 mmol) of 4-bromo-*N,N*-diphenylaniline were mixed with 2.13 g (8.4 mmol, 1.5 equiv) of B_2pin_2, 0.41 g (0.56 mmol, 10% mol) of $Pd(dppf)Cl_2$ and 1.65 g (16.8 mmol, 3 equiv) of potassium acetate. The reagents were purged with argon three times and 20 mL of 1,4-dioxane anhydrous were added. The reaction was left stirring for 12 hours at 90°C using an oil bath and under argon atmosphere. When completed, the reaction mixture was filtered with celite and washed with ethyl acetate (3x20mL). The crude product was recovered in a 100 mL round-bottom flask and after evaporating the solvent, it was purified by a silica gel column chromatography using *n*-hexane/THF (20:1) as mobile phase. After purification, 1.56 g (4.20 mmol, 75% yield) of **6** were obtained in the form of a white powder. Mp: 92-94 °C. R_f = 0.36 (n-hexane/THF = 20:1 v/v. ^{1}H NMR (400 MHz, $CDCl_3$) $\delta_{(ppm)}$: 7.70-7.63 (m, 2H), 7.29-7.22 (m, 4H), 7.11 (m, 2H), 7.09 (dd, *J* = 2.1, 1.0 Hz, 2H), 7.05-7.01 (m, 4H), 1.33 (s, 12H). ^{13}C {^{1}H} NMR (101 MHz, $CDCl_3$) $\delta_{(ppm)}$: 151.1, 147.9 (2C), 136.3 (2C), 129.8 (4C), 125.4 (5C), 123.8 (2C), 122.3 (2C), 84.0 (2C), 25.3 (4C). HRMS (ESI) m/z calcd. for ($C_{24}H_{27}BNO_2$) $[M+H]^+$ 372.2129, found 372.2132.

- ***Synthesis of 1-bromo-2,4-bis(dodecycloxy)benzene* SI1*:***

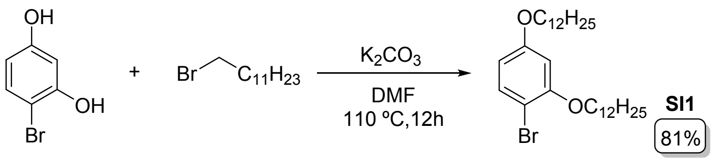

In 250 mL round-bottom flask 2.04 g (11 mmol) of 4-bromoresorcinol were mixed with 6.4 g (44 mmol, 4.2 equiv) of K_2CO_3 and 80 mL of DMF. The mixture was heated up to 90°C and was left stirring for 1 hour. Next, 6.84 mL (28.56 mmol, 2.6 equiv) of 1-bromododecane were added and the reaction was left stirring overnight at 110 °C using an oil bath. When the reaction was completed, the mixture was extracted with EtOAc (3x20mL) and washed with brine (3x15mL). The organic phase was dried over anhydrous magnesium sulphate and filtered. The solvent was evaporated and the crude product was purified by a silica gel column chromatography using a mixture of *n*-hexane/THF 25:1. After purification, 4.2 g (7.86 mmol, 81 % yield) of 1-bromo-2,4-bis(dodecycloxy)benzene **SI1** were obtained as a yellow oil. R_f = 0.77 (*n*-hexane/THF = 25:1 v/v). ^{1}H NMR (400 MHz, $CDCl_3$) $\delta_{(ppm)}$: 7.37 (d, *J* = 8.7 Hz, 1H), 6.46 (d, *J* = 2.7 Hz, 1H), 6.36 (dd, *J* = 8.7, 2.7 Hz, 1H), 3.98 (t, *J* = 6.5 Hz, 2H), 3.91 (t, *J* = 6.6 Hz, 2H), 1.88-1.79 (m, 2H), 1.79-1.71 (m, 2H), 1.51-1.40 (m, 2H), 1.39-1.21 (m, 34H), 0.92-0.84 (t, *J* = 6.9Hz 6H). ^{13}C {^{1}H} NMR (126 MHz, $CDCl_3$) $\delta_{(ppm)}$: 159.5, 156.0, 132.9, 106.3, 102.7, 101.3, 69.0, 68.2, 31.8 (2C), 29.6 (4C), 29.5 (4C), 29.4 (4C), 29.2, 29.1, 25.9, 25.8, 22.6, 14.0 (2C). HRMS (ESI) m/z calcd. for ($C_{30}H_{53}BrNaO_2$) $[M+Na]^+$ 547.3127, found 547.3123.

- ***Synthesis of 2-(2,4-bis(dodecyloxy)phenyl)-4,4,5,5-tetramethyl-1,3,2-dioxaborolane 16:***

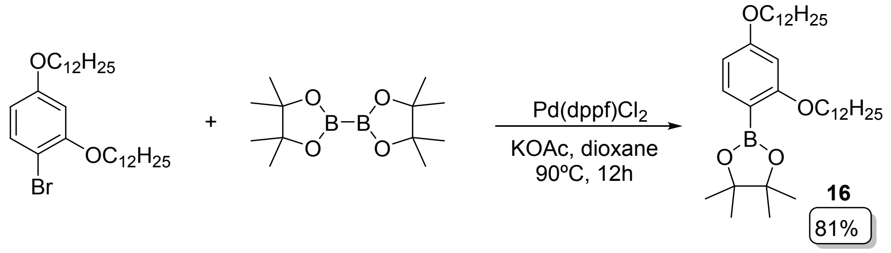

In 250 mL round-bottom flask 3.27 g (6.21 mmol) of 1-bromo-2,4-bis(dodecycloxy)benzene were mixed with 2.37 g (9.33 mmol, 1.5 equiv) of bis(pinacolato)diboron (B_2pin_2), 0.45 g (0.60 mmol, 10% mol) of Pd(dppf)Cl_2 and 1.83 g (18.66 mmol, 3 equiv) of potassium acetate. The reagents were purged with argon three times and 20 mL of 1,4-dioxane anhydrous were added. The reaction was left stirring for 12 hours at 90°C using an oil bath and under argon atmosphere. When completed, the reaction mixture was filtered with celite and washed with ethyl acetate (3x20mL). The crude product was recovered in a 100 mL round-bottom flask and after evaporating the solvent, it was purified by a silica gel column chromatography using *n*-hexane/THF (20:1) as mobile phase. After purification, 2.85 g (4.98 mmol, 80% yield) of **16** were obtained as a pale-yellow oil. R_f = 0.36 (*n*-hexane/THF = 20:1 v/v). ^{1}H NMR (500 MHz, $CDCl_3$) $\delta_{(ppm)}$: 7.57 (d, *J* = 8.2 Hz, 1H), 6.44 (, dd, *J* = 8.2, 2.1 Hz, 1H), 6.38 (d, *J* = 2.1 Hz, 1H), 3.94 (dt, *J* = 9.6, 6.4 Hz, 4H), 1.77 (dt, *J* = 8.2, 6.6 Hz, 6H), 1.32 (s, 12H), 1.29-1.24 (m, 34H), 0.88 (t, *J* = 6.9 Hz, 6H). ^{13}C {^{1}H} NMR (101 MHz, $CDCl_3$) $\delta_{(ppm)}$: 165.7, 163.4, 138.1, 106.8, 105.9, 99.2, 68.5, 68.2, 32.1 (4C), 29.8 (14C), 29.5 (2C), 29.4 (2C), 26.2 (2C), 22.8 (2C), 14.3 (2C). HRMS (ESI) m/z calcd. for ($C_{36}H_{65}BrNaO_4$) $[M+Na]^+$ 595.4874, found 595.4870.

3. Characterization data for the side product 13'

Compound **13'** was obtained as a yellow oil in 27 % yield: ^{1}H NMR (400 MHz, $CDCl_3$) $\delta_{(ppm)}$: 9.93 (s, 1H), 8.15 (dt, J = 8.3, 1.9 Hz, 2H), 7.52 (ddt, J = 13.6, 8.4, 1.7 Hz, 3H), 7.28 (dd, J = 7.3, 1.7 Hz, 1H), 6.84 (td, J = 7.6, 1.8 Hz, 1H), 6.21 (br.s, 2H), 4.41 (q, J = 7.1 Hz, 2H), 1.42 (t, J = 7.1 Hz, 3H). ^{13}C {^{1}H} NMR (126 MHz, $CDCl_3$) $\delta_{(ppm)}$: 194.1, 166.3, 147.2, 142.5, 136.1, 136.0, 130.5, 130.0, 129.2, 127.3, 119.1, 116.3, 61.2, 14.4.

4. Experimental details and characterization data of photosensitiser 19

- ***Synthesis of 4-(benzo[c]isoxazol-7-yl)-N,N-diphenylaniline 7:***

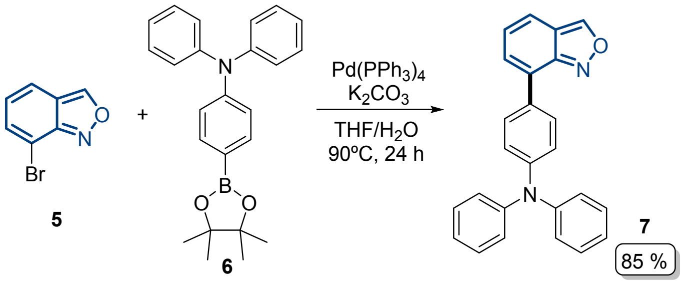

In a 25 mL glass reactor vessel, anthranil **5** (0.47 g, 2.38 mmol) was mixed with 1.50 g (4.05 mmol, 1.7 equiv) of **6**, 0.14 g (0.12 mmol, 5% mol) of $Pd(PPh_3)_4$ and 0.99 g (7.15 mmol, 3 equiv) of K_2CO_3. The reagents were purged with argon three times and 8 mL of THF and 4 mL of deionized water were added into the vessel. The reaction was left stirring for 24 hours at 90°C using a heating block and under argon atmosphere. The resulting reaction mixture was extracted with EtOAc (3x20mL) and washed with a saturated brine solution (3x15mL). Next, the organic phase was dried over anhydrous magnesium sulphate and filtered. The crude product was purified by a silica gel column chromatography using a 5:1 mixture of *n*-hexane and THF as eluent. After purification, 0.73 g (2.01 mmol, 85% yield) of 4-(benzo[*c*]isoxazol-7-yl)-*N,N*-diphenylaniline **7** were obtained as a bright yellow solid. Mp: 134-136 °C. R_f = 0.17 (*n*-hexane/THF = 10:1 v/v). IR (ATR, ν_{max}, cm^{-1}): 1505 $\nu_{C-O,}$ 1495 $\nu_{N-O,}$ 1270 ν_{C-N}. ^{1}H NMR (500 MHz, $CDCl_3$) $\delta_{(ppm)}$: 9.19 (s, 1H), 7.94-7.86 (m, 2H), 7.50 (dd, J = 8.8, 0.9 Hz, 1H), 7.43 (dd, J = 6.8, 0.9 Hz, 1H), 7.33-7.24 (m, 4H), 7.22-7.14 (m, 6H), 7.11 (dd, J = 8.7, 6.8 Hz, 1H), 7.06 (t, J = 7.3, 2H). ^{13}C {^{1}H} NMR (126 MHz, $CDCl_3$) $\delta_{(ppm)}$: 155.5, 154.8, 148.1, 147.6 (2C), 130.4, 129.4 (6C), 128.5, 127.7 (2C), 125.3, 124.9 (4C), 123.3 (2C), 119.5, 118.0 (2C). HRMS (ESI) m/z calcd. for ($C_{25}H_{19}N_2O$) [M+H]$^+$ 363.1492, found 363.1490.

- ***Synthesis of ethyl 4-(7-(4-(diphenylamino)phenyl)benzo[c]isoxazol-3-yl)benzoate 11:***

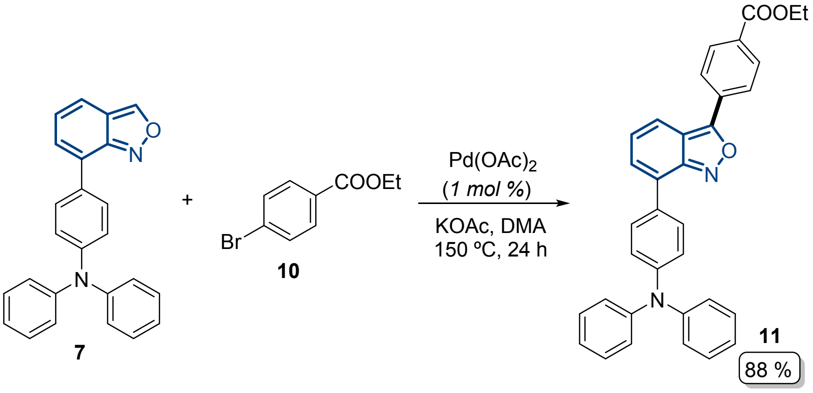

In a 25 mL glass reactor vessel, a mixture of anthranil **7** (0.63 g, 1.74 mmol, 1.5 equiv), ethyl-4-bromobenzoate **10** (0.19 mL, 1.15 mmol) and KOAc (0.23 g, 2.31 mmol, 2 equiv) were

dissolved with anhydrous DMA under an argon atmosphere. Next, 2.60 mg (0.01 mmol, 1% mol) of $Pd(OAc)_2$ were added and the reaction was heated slowly until 150 °C using a heating block. The reaction mixture was left stirring for 24 hours under argon atmosphere. Then, the crude product was purified through a silica gel column chromatography using as eluent a 3:1 mixture of *n*-hexane/THF. After purification, 0.52 g (1.02 mmol, 88% yield) of ethyl 4-(7-(4-(diphenylamino)phenyl)benzo[*c*]isoxazol-3-yl)benzoate **11** was obtained in the form of an orange oil. R_f = 0.33 (*n*-hexane/THF = 5:1 v/v). IR (ATR, ν_{max}, cm^{-1}): 1680 $\nu_{C=O,}$ 1505 $\nu_{C=C,}$ 1270 $\nu_{C\text{-}O.}$ ^1H NMR (500 MHz, CD_2Cl_2) $\delta_{(ppm)}$: 8.23 (d, J = 8.7 Hz, 2H,), 8.13 (d, J = 8.7 Hz, 2H,), 7.91 (d, J = 8.8 Hz, 2H,), 7.81 (dd, J = 8.8, 0.9 Hz, 1H,), 7.49 (dd, J = 6.9, 0.9 Hz, 1H,), 7.36 – 7.27 (m, 4H,), 7.22 (dd, J = 8.8, 6.9 Hz, 1H,), 7.19 – 7.14 (m, 6H,), 7.08 (tt, J = 7.0, 1.2 Hz, 2H,), 4.41 (q, J = 7.1 Hz, 2H,), 1.42 (t, J = 7.1 Hz, 3H,). ^{13}C {^1H} NMR (126 MHz, CD_2Cl_2) $\delta_{(ppm)}$: 165.6, 163.2, 157.0, 148.1, 147.5(2C), 131.9, 131.7, 130.3 (2C), 130.0, 129.4 (2C), 129.3 (4C), 128.8, 127.6, 126.3 (2C), 126.2, 124.8 (4C), 123.3 (2C), 122.8 (2C), 118.5, 116.4, 61.3, 14.1. HRMS (ESI) m/z calcd. for [$C_{34}H_{27}N_2O_3$] $[M+H]^+$ 511.2016, found 511.2012.

- ***Synthesis of ethyl 4-(7-(4-(bis(4-bromophenyl)amino)phenyl)benzo[c]isoxazol-3-yl)benzoate 14:***

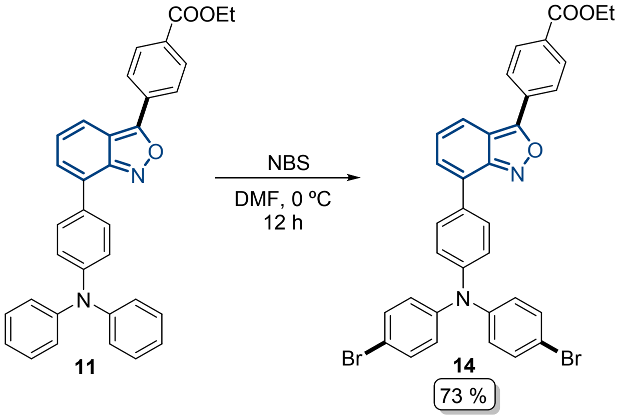

In 100 mL round-bottom flask, 0.44 g (0.87 mmol) of benzoate **11** were mixed with 0.40 g (2.25 mmol, 2.6 equiv) of NBS in 30 mL of DMF. The reaction mixture was stirred for 12 hours at 0°C. Then, brine was added into the reactor and the mixture was left in the freezer for 1 hour to precipitate. Next, the mixture was filtered and washed with EtOAc (3x20mL) and the crude product was collected in a 100 mL round bottom flask. The solvent was evaporated and the crude was purified by a silica gel chromatographic column using a mixture of *n*-hexane/THF 5:1 as eluent. After purification, 0.42 g (0.63 mmol, 73% yield) of ethyl 4-(7-(4-(bis(4-bromophenyl)amino)phenyl)benzo[*c*]isoxazol-3-yl)benzoate **14** were obtained as an orange oil. R_f = 0.43 (*n*-hexane/THF = 5:1 v/v). IR (ATR, ν_{max}, cm^{-1}): 1700 $\nu_{C=O}$, 1490 $\nu_{N\text{-}O,}$ 1280 $\nu_{C\text{-}O.}$ ^1H NMR (400 MHz, CD_2Cl_2) $\delta_{(ppm)}$: 8.27 (d, J = 8.8 Hz, 2H), 8.18 (d, J = 8.8 Hz, 2H), 7.98 (d, J = 8.9 Hz, 2H), 7.87 (dd, J = 8.8, 0.9 Hz, 1H), 7.53 (dd, J = 6.8, 0.9 Hz, 1H,), 7.47 – 7.42 (m, 4H), 7.27 (dd, J = 8.8, 6.8 Hz, 1H,), 7.21 (d, J = 8.6 Hz, 2H), 7.08 (d, J = 8.9 Hz, 4H), 4.45 (q, J = 7.1 Hz, 2H), 1.46 (t, J = 7.1 Hz, 3H). ^{13}C {^1H} NMR (101 MHz, CD_2Cl_2) $\delta_{(ppm)}$: 166.0, 163.7, 157.4, 147.5 (2C), 146.7, 132.8 (4C), 132.3, 132.2, 131.7, 130.7 (2C), 130.1 (2C), 128.9, 128.3, 126.7 (2C), 126.5, 126.4 (4C), 124.1 (2C), 119.3, 116.8, 116.3 (2C), 61.8, 14.5. HRMS (ESI) m/z calcd. for ($C_{34}H_{25}Br_2N_2O_3$) $[M+H]^+$ 667.0226, found 667.0222.

- ***Synthesis of ethyl 4-(7-(4-(bis(2',4'-bis(dodecycloxy)-[1,1'-biphenyl]-4-yl)amino) phenyl)benzo[c]isoxazole-3-yl)benzoate 17***

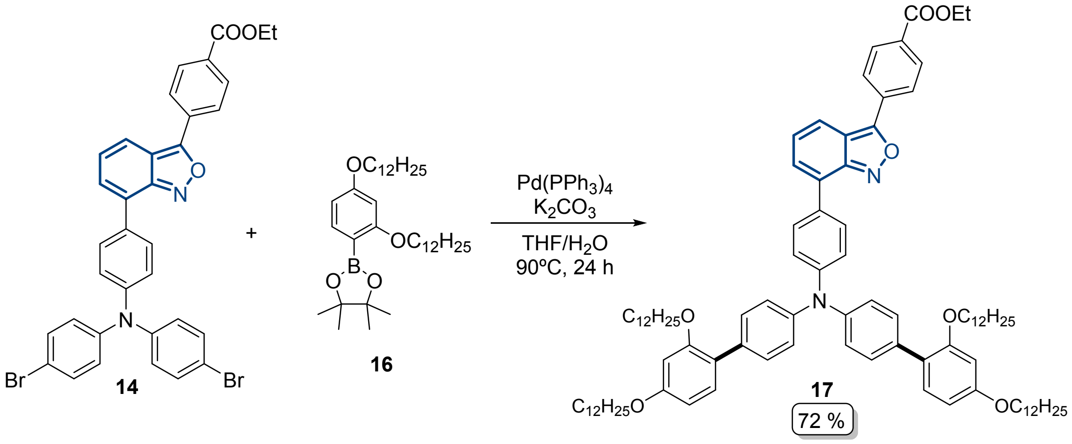

In a 25 mL glass reactor vessel, benzoate **14** (0.40 g, 0.60 mmol), boronate **16** (0.90 g, 1.57 mmol, 2.6 equiv), 0.07 g of $Pd(PPh_3)_4$ (0.06 mmol, 5% mol) and K_2CO_3 (0.50 g, 3.63 mmol, 6 equiv) added and the system was purged with argon three times. Then, 10 mL of THF and 5 mL of deionized water were added into the vessel. The reaction was left stirring for 24 hours at 90°C using a heating block and under argon atmosphere. The resulting reaction mixture was extracted with EtOAc (3x20mL) and washed with a saturated brine solution (3x15mL). Next, the organic phase was dried over anhydrous magnesium sulphate and filtered. The crude product was purified by a silica gel chromatographic column using a 10:1 mixture of *n*-hexane and THF as eluent. After purification, 0.316 g (0.43 mmol, 72% yield) of 4-(7-(4-(bis(2',4'-bis(dodecycloxy)-[1,1'-biphenyl]-4-yl)amino) phenyl)benzo[*c*]isoxazole-3-yl)benzoate **17** were obtained as an orange oil. R_f = 0.60 (*n*-hexane/THF = 10:1 v/v). IR (ATR, ν_{max}, cm^{-1}): 2850 $\nu_{C\text{-}H,}$ 1700 $\nu_{C=O,}$ 1490 $\nu_{N\text{-}O,}$ 1290 $\nu_{C\text{-}O.}$ ^{1}H NMR (400 MHz, $CDCl_3$) $\delta_{(ppm)}$: 8.25 (d, J = 8.5 Hz, 2H), 8.13 (d, J = 8.5 Hz, 2H), 7.93 (d, J = 8.7 Hz, 1H), 7.79 (d, J = 8.8 Hz, 1H), 7.52 – 7.43 (m, 5H), 7.29 (dd, J = 8.9, 6.0 Hz, 4H), 7.26 – 7.19 (m, 5H), 6.55 (dd, J = 6.0, 2.4 Hz, 4H), 4.44 (d, J = 7.1 Hz, 2H), 4.05 – 3.90 (m, 8H), 1.89 – 1.72 (m, 8H), 1.45 (t, J = 7.1 Hz, 3H), 1.35 – 1.19 (m, 73H), 0.87 (dt, J = 18.3, 6.9 Hz, 12H). ^{13}C {^{1}H} NMR (101 MHz, $CDCl_3$) $\delta_{(ppm)}$: 165.8, 163.0, 159.5, 157.1, 157.0, 148.2, 145.5, 133.3, 132.1, 131.5, 130.8, 130.4, 130.2, 129.7, 129.3, 129.2, 127.4, 126.3, 124.1, 123.0, 118.2, 116.4, 105.3, 100.4, 68.4, 68.1, 61.3, 31.9, 31.9, 29.6, 29.6, 29.6, 29.6, 29.4, 29.3, 29.3, 29.3, 29.1, 26.1, 22.7, 22.6, 14.3, 14.1, 14.1. HRMS (MALDI) m/z calcd. for [$C_{94}H_{130}N_2O_7$] 1398.9878, found 1398.9887.

- ***Synthesis of 4-(7-(4-(bis(2',4'-bis(dodecycloxy)-[1,1'-biphenyl]-4-yl)amino) phenyl)benzo[c]isoxazole-3-yl)benzoic acid 19***

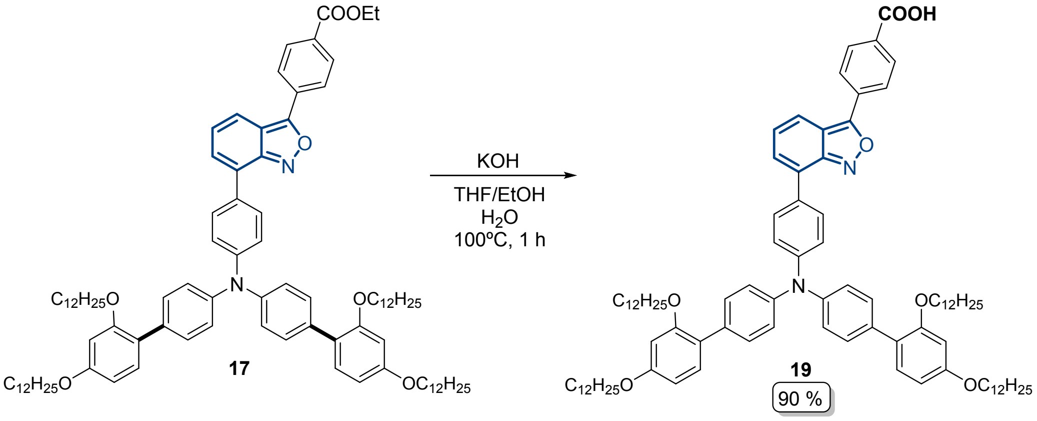

In a 25 mL glass reactor vessel, 0.32 g (0.23 mmol) of benzoate **17**, were mixed with 0.12 g (2.21 mmol, 9.6 equiv) of KOH and to this mixture 3 mL of THF, 1 mL of H_2O and 1 mL of EtOH were added in one portion. The system was stirred for 1 hour at 100°C using a heating block. After the reaction was completed, the mixture was poured into a 100mL round-bottom flask and the solvent was removed under vacuum. Then, 20 mL of HCl solution (5 M) were added in order to neutralize the mixture and it was left in the freezer for 1 hour to precipitate. The crude product was extracted and washed with DCM (3x15mL). The extra solvent was evaporated and the crude was purified by a silica gel chromatographic column. The pure product was collected using a mixture of DCM/MeOH 10:1 containing four drops of acetic acid. After purification 0.28 g (0.20 mmol, 94% yield) of 4-(7-(4-(bis(2',4'-bis(dodecycloxy)-[1,1'-biphenyl]-4-yl)amino) phenyl)benzo[*c*]isoxazole-3-yl)benzoic acid **19** were obtained as a red oil. R_f = 0.63 (DCM/MeOH 10:1 v/v). IR (ATR, ν_{max}, cm^{-1}): 2920 $\nu_{C\text{-}H,}$ 2900 $\nu_{O\text{-}H,}$ 1700 $\nu_{C=O,}$ 1610 $\nu_{C=C}$, 1500 $\nu_{N\text{-}O,}$ 1290 $\nu_{C\text{-}O.}$ ^1H NMR (500 MHz, CD_2Cl_2) $\delta_{(ppm)}$: 8.31 (d, J = 8.5 Hz, 2H), 8.19 (d, J = 8.5 Hz, 2H), 7.95 (d, J = 8.7 Hz, 2H), 7.83 (d, J = 8.8 Hz, 1H), 7.52 (d, J = 6.8 Hz, 1H), 7.49 (d, J = 8.6 Hz, 4H), 7.28 – 7.25 (m, 5H), 7.21 (d, J = 8.7 Hz, 4H), 6.55 (m, 4H), 3.98 (td, J = 6.5, 4.7 Hz, 8H), 1.78 (m, 8H), 1.37-1.52 (m, 73H), 0.89 (t, J = 6.9 Hz, 6H), 0.84 (t, J = 6.9 Hz, 6H). ^{13}C {^1H} NMR (126 MHz, CD_2Cl_2) $\delta_{(ppm)}$: 170.1, 163.3, 160.2 (2C), 157.4 (2C), 148.7, 146.0 (3C), 134.1, 131.4 (2C), 131.2 (2C), 130.7 (5C), 130.2, 129.8 (2C), 129.4, 127.9, 126.9 (4C), 124.6 (4C), 123.1 (6C), 117.0, 105.9 (2C), 100.7 (2C), 68.9 (2C), 68.6 (2C), 32.3 (4C), 30.1 (18C), 29.8 (10), 26.5 (4C), 23.1 (4C), 14.3 (4C). HRMS (MALDI) m/z calcd. for [$C_{92}H_{126}N_2O_7$], 1370.9566, found 1370.9532.

5. Experimental details and characterization data of photosensitiser 20

- ***Synthesis of ethyl 4-(benzo[c]isoxazol-7-yl)benzoate 9:***

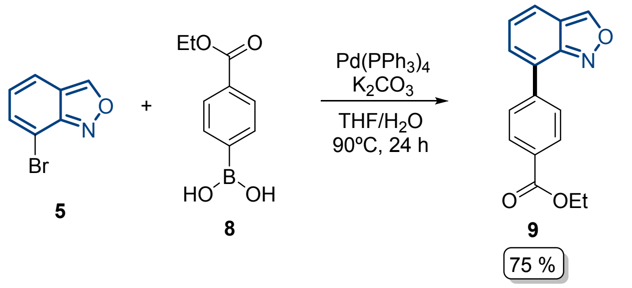

In a 25 mL glass reactor vessel, anthranil **5** (0.40 g, 2.05 mmol) was mixed with 0.52 g (2.68 mmol, 1.3 equiv) of **8**, 0.12 g (0.102 mmol, 5% mol) of $Pd(PPh_3)_4$ and 0.85 g (6.15 mmol, 3 equiv) of K_2CO_3. The reagents were purged with argon three times and 8 mL of THF and 4 mL of deionized water were added into the vessel. The reaction was left stirring for 24 hours at 90°C using a heating block and under argon atmosphere. The resulting reaction mixture was extracted with EtOAc (3x20mL) and washed with a saturated brine solution (3x15mL). Next, the organic phase was dried over anhydrous magnesium sulphate and filtered. The crude product was purified by a silica gel column chromatography using a 2:1 mixture of *n*-hexane and THF as eluent. After purification, 0.41 g (1.53 mmol, 75% yield) of ethyl 4-(benzo[*c*]isoxazol-7-yl)benzoate **9** were obtained as a yellow solid. Mp: 148-150 °C. R_f = 0.55 (*n*-hexane/THF = 2:1 v/v). IR (ATR, ν_{max}, cm^{-1}): 1506 $\nu_{C\text{-}O,}$ 1495 $\nu_{N\text{-}O,}$ 1272 $\nu_{C\text{-}N}$. ^1H NMR (500 MHz, $CDCl_3$) $\delta_{(ppm)}$: 9.24 (s, 1H), 8.17-8.14 (m, 2H), 8.08-8.05 (m, 2H), 7.60 (dd, J = 8.8, 0.9 Hz, 1H), 7.53 (dd, J = 6.8, 0.9 Hz, 1H), 7.14 (dd, J = 8.7, 6.7 Hz, 1H), 4.41 (q, J = 7.1 Hz, 2H), 1.42 (t, J = 7.1 Hz, 3H). ^{13}C {^1H} NMR (126 MHz, $CDCl_3$) $\delta_{(ppm)}$: 166.5, 155.2, 141.0,

130.2, 129.9 (2C), 129.5, 128.5 (2C), 127.8, 125.0, 119.8, 119.4, 61.1, 14.4. HRMS (ESI) m/z calcd. for ($C_{16}H_{14}NO_3$) $[M+H]^+$ 268.0968, found 268.0971.

- ***Synthesis of ethyl 4-(3-(4-(diphenylamino)phenyl)benzo[c]isoxazol-7-yl)benzoate 13:***

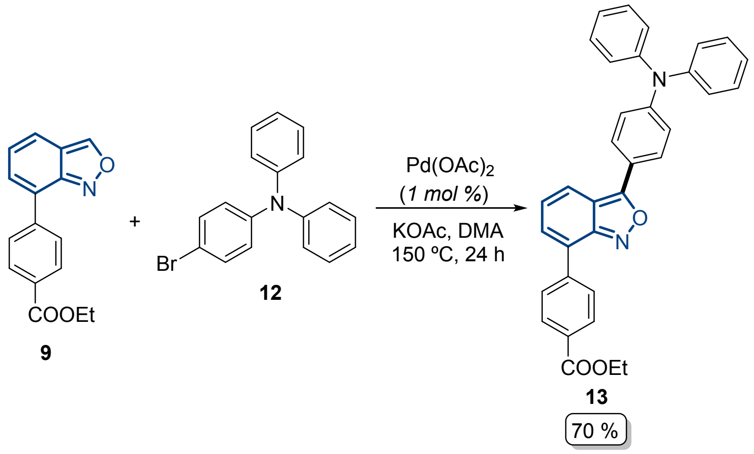

In a 25 mL glass reactor vessel, a mixture of anthranil **9** (0.37 g, 1.38 mmol, 1.5 equiv), **12** (0.30 g, 0.925 mmol) and KOAc (0.181 g, 1.85 mmol, 2 equiv) were dissolved with anhydrous DMA under an argon atmosphere. Next, 2.07 mg (0.092 mmol, 1% mol) of $Pd(OAc)_2$ were added and the reaction was heated slowly until 150 °C using a heating block. The reaction mixture was left stirring for 24 hours under argon atmosphere. Then, the crude product was purified through a silica gel column chromatography using as eluent a 10:3 mixture of *n*-hexane/THF. After purification, 0.33 g (0.647 mmol, 70% yield) of ethyl 4-(3-(4-(diphenylamino)phenyl)benzo[*c*]isoxazol-7-yl)benzoate **13** was obtained in the form of an orange solid. R_f = 0.3 (*n*-hexane/THF = 10:3 v/v). IR (ATR, ν_{max}, cm^{-1}): 1680 $\nu_{C=O,}$ 1504 $\nu_{C=C,}$ 1271 $\nu_{C\text{-}O.}$ ^1H NMR (400 MHz, CD_2Cl_2) $\delta_{(ppm)}$: 8.16 – 8.10 (m, 2H), 7.91 – 7.88 (m, 1H), 7.85 (dd, J = 8.8, 0.9 Hz, 1H), 7.56 (dd, J = 6.8, 0.9 Hz, 1H), 7.35 (dd, J = 8.6, 7.3 Hz, 2H), 7.21 – 7.12 (m, 6H), 4.40 (q, J = 7.2 Hz, 1H), 1.42 (t, J = 7.1 Hz, 3H). ^{13}C {^1H} NMR (101 MHz, CD_2Cl_2) $\delta_{(ppm)}$: 166.5, 165.6, 156.9, 150.3, 147.1, 141.5, 130.4, 130.0 (4C), 129.9 (2C), 129.7, 128.8 (2C), 128.0, 127.7, 126.1 (4C), 124.7, 124.7, 121.8, 121.4, 121.1, 115.1, 61.3, 14.5. HRMS (ESI) m/z calcd. for ($C_{34}H_{27}N_2O_3$) $[M+H]^+$ 511.2016, found 511.2013.

- ***Synthesis of ethyl 4-(3-(4-(bis(4-bromophenyl)amino)phenyl)benzo[c]isoxazol-7-yl)benzoate 15:***

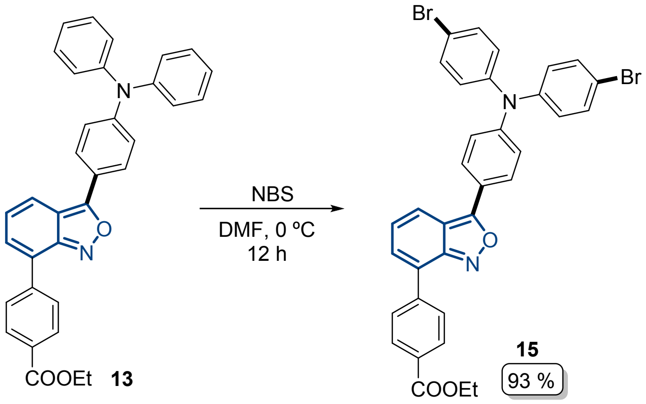

In 100 mL round-bottom flask, 0.39 g (0.58 mmol) of benzoate **13** were mixed with 0.27 g (1.51 mmol, 2.6 equiv) of NBS in 30 mL of DMF. The reaction mixture was stirred for 12 hours at 0°C. Then, brine was added into the reactor and the mixture was left in the freezer for 1 hour to precipitate. Next, the mixture was filtered and washed with EtOAc (3x20mL)

and the crude product was collected in a 100 mL round bottom flask. The solvent was evaporated and the crude was purified by a silica gel chromatographic column using a mixture of *n*-hexane/THF 10:1 as eluent. After purification, 0.36 g (0.538 mmol, 93% yield) of ethyl 4-(3-(4-(bis(4-bromophenyl)amino)phenyl)benzo[*c*]isoxazol-7-yl)benzoate **15** were obtained as a reddish solid. R_f = 0.40 (*n*-hexane/THF = 0:1 v/v). IR (ATR, ν_{max}, cm^{-1}): 1698 $\nu_{C=O}$, 1488 $\nu_{N\text{-}O}$, 1281 $\nu_{C\text{-}O}$. ^{1}H NMR (400 MHz, CD_2Cl_2) $\delta_{(ppm)}$: 8.13 (q, *J* = 8.7 Hz, 4H), 7.93 (d, *J* = 8.8 Hz, 2H), 7.85 (d, *J* = 8.0 Hz, 1H), 7.57 (d, *J* = 6.3 Hz, 1H), 7.45 (d, *J* = 8.8 Hz, 4H), 7.21 – 7.14 (m, 3H), 7.06 (d, *J* = 8.8 Hz, 4H), 4.39 (q, *J* = 7.2 Hz, 2H), 1.42 (t, *J* = 7.1 Hz, 3H). ^{13}C {^{1}H} NMR (101 MHz, CD_2Cl_2) $\delta_{(ppm)}$: 166.5, 165.2, 156.9, 149.3, 146.0 (2C), 141.4, 133.1 (4C), 130.5, 130.0 (2C), 129.8, 128.8 (2C), 128.2 (2C), 127.9, 127.2 (4C), 125.0, 123.0 (2C), 122.4, 121.2, 117.3, 61.4, 14.5. HRMS (ESI) m/z calcd. for ($C_{34}H_{25}Br_2N_2O_3$) $[M+H]^+$ 667.0226, found 667.0223.

- ***Synthesis of ethyl 4-(3-(4-(bis(2',4'-bis(dodecyloxy)-[1,1'-biphenyl]-4-yl)amino)phenyl)benzo[c]isoxazol-7-yl)benzoate 18***

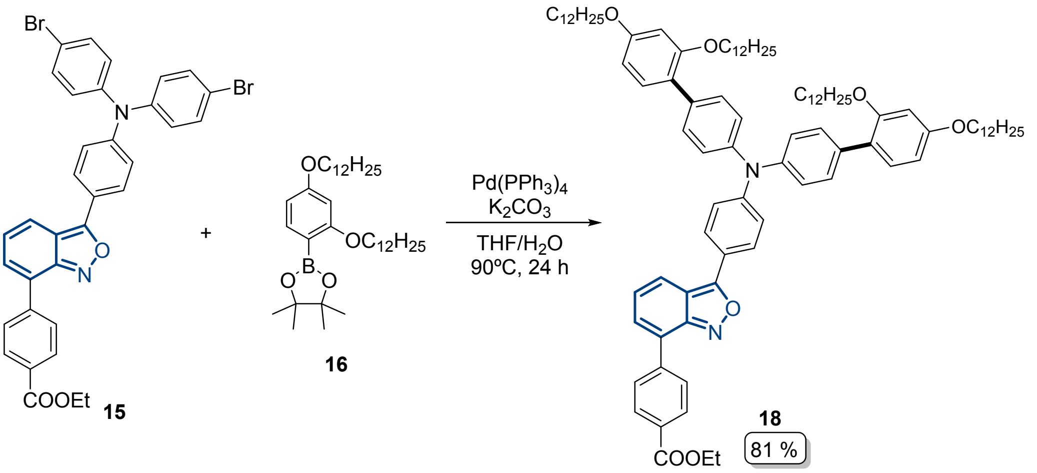

In a 25 mL glass reactor vessel, benzoate **15** (0.2 g, 0.29 mmol), boronate **16** (0.42 g, 0.74 mmol, 2.5 equiv), 52 mg of $Pd(PPh_3)_4$ (0.044 mmol, 15% mol) and K_2CO_3 (0.24 g, 1.79 mmol, 6 equiv) added and the system was purged with argon three times. Then, 10 mL of THF and 5 mL of deionized water were added into the vessel. The reaction was left stirring for 24 hours at 90°C using a heating block and under argon atmosphere. The resulting reaction mixture was extracted with EtOAc (3x20mL) and washed with a saturated brine solution (3x15mL). Next, the organic phase was dried over anhydrous magnesium sulphate and filtered. The crude product was purified by a silica gel chromatographic column using a 15:1 mixture of *n*-hexane and THF as eluent. After purification, 0.334 g (0.238 mmol, 81% yield) of ethyl 4-(3-(4-(bis(2',4'-bis(dodecyloxy)-[1,1'-biphenyl]-4-yl)amino)phenyl)benzo[*c*]isoxazol-7-yl)benzoate **18** were obtained as an orange oil. R_f = 0.43 (*n*-hexane/THF = 15:1 v/v). IR (ATR, ν_{max}, cm^{-1}): 2851 $\nu_{C\text{-}H}$, 1689 $\nu_{C=O}$, 1494 $\nu_{N\text{-}O}$, 1291 $\nu_{C\text{-}O}$. ^{1}H NMR (400 MHz, CD_2Cl_2) $\delta_{(ppm)}$: 8.17 – 8.11 (m, 4H), 7.96 – 7.92 (m, 2H), 7.88 (dd, *J* = 8.8, 0.9 Hz, 1H), 7.58 (dd, *J* = 6.8, 0.9 Hz, 1H), 7.56 – 7.52 (m, 4H), 7.29 – 7.22 (m, 8H), 7.16 (dd, *J* = 8.8, 6.8 Hz, 1H), 6.56 (d, *J* = 7.3 Hz, 4H), 4.40 (q, *J* = 7.1 Hz, 2H), 3.99 (td, *J* = 6.5, 2.4 Hz, 8H), 1.78 (ddd, *J* = 14.2, 11.7, 6.6 Hz, 8H), 1.37 – 1.19 (m, 75H), 0.92 – 0.88 (m, 6H), 0.86 – 0.82 (m, 6H). ^{13}C {^{1}H} NMR (101 MHz, CD_2Cl_2) $\delta_{(ppm)}$: 166.6, 165.7, 160.3, 157.4, 156.9, 145.2, 141.6, 135.1, 131.2, 130.9, 130.4, 130.0, 129.7, 128.8, 128.0, 125.8, 125.4, 124.6, 122.9, 121.8, 121.5, 120.9, 115.1, 105.9, 100.6, 68.8, 68.6, 61.4, 32.3, 32.3, 30.1, 30.0, 30.0, 30.0, 29.8, 29.8, 29.7, 29.7, 29.6, 26.5, 26.5, 23.1, 23.1, 14.5, 14.3, 14.3. HRMS (MALDI) m/z calcd. for [$C_{94}H_{130}N_2O_7$] 1398.9878, found 1398.9888.

- ***Synthesis of 4-(3-(4-(bis(2',4'-bis(dodecyloxy)-[1,1'-biphenyl]-4-yl)amino)phenyl)benzo[c]isoxazol-7-yl)benzoic acid 20***

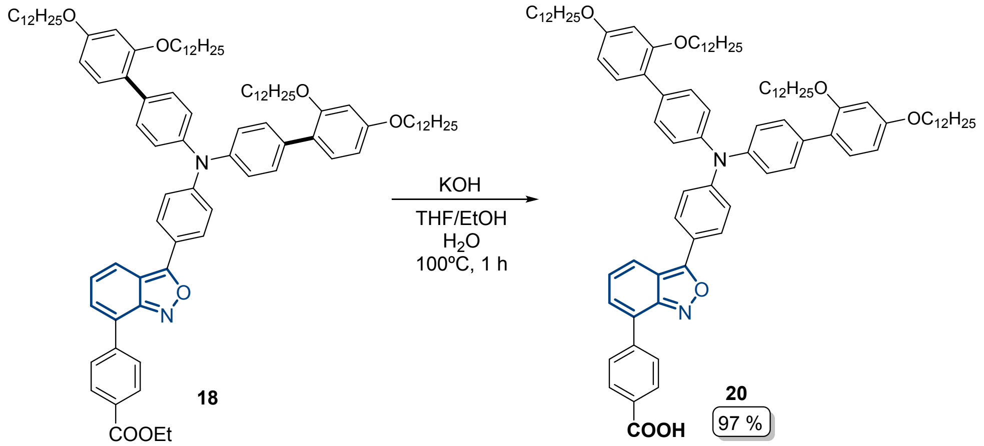

In a 25 mL glass reactor vessel, 0.23 g (0.167 mmol) of benzoate **18**, were mixed with 90 mg (1.6 mmol, 9.6 equiv) of KOH and to this mixture 3 mL of THF, 1 mL of H_2O and 1 mL of EtOH were added in one portion. The system was stirred for 1 hour at 100°C using a heating block. After the reaction was completed, the mixture was poured into a 100mL round-bottom flask and the solvent was removed under vacuum. Then, 20 mL of HCl solution (5 M) were added in order to neutralize the mixture and it was left in the freezer for 1 hour to precipitate. The crude product was extracted and washed with DCM (3x15mL). The extra solvent was evaporated and the crude was purified by a silica gel chromatographic column. The pure product was collected using a mixture of DCM/MeOH 10:1 containing four drops of acetic acid. After purification 0.21 g (0.153 mmol, 95% yield) of 4-(3-(4-(bis(2',4'-bis(dodecyloxy)-[1,1'-biphenyl]-4-yl)amino)phenyl)benzo[*c*]isoxazol-7-yl)benzoic acid **20** were obtained as a red solid. R_f = 0.52 (DCM/MeOH 10:1 v/v). IR (ATR, ν_{max}, cm^{-1}): 2918 $\nu_{C-H,}$ 2901 $\nu_{O-H,}$ 1680 $\nu_{C=O,}$ 1611 $\nu_{C=C}$, 1498 $\nu_{N-O,}$ 1291 $\nu_{C-O.}$ ^{1}H NMR (400 MHz, CD_2Cl_2) $\delta_{(ppm)}$: 8.26 – 8.16 (m, 4H), 7.94 (d, *J* = 8.9 Hz, 2H), 7.88 (d, *J* = 9.2 Hz, 1H), 7.60 (d, *J* = 6.5 Hz, 1H), 7.55 (d, *J* = 8.8 Hz, 4H), 7.26 (dd, *J* = 12.1, 8.7 Hz, 8H), 7.16 (dd, *J* = 8.8, 6.8 Hz, 1H), 6.56 (d, *J* = 8.1 Hz, 4H), 4.00 (td, *J* = 6.6, 1.9 Hz, 8H), 1.78 (ddd, *J* = 14.3, 11.4, 6.7 Hz, 8H), 1.52 – 1.40 (m, 12H), 1.32 – 1.19 (m, 63H), 0.93 – 0.88 (m, 6H), 0.87 – 0.82 (m, 6H). ^{13}C {^{1}H} NMR (101 MHz, CD_2Cl_2) $\delta_{(ppm)}$: 165.7, 160.3, 157.4, 156.8, 150.5, 145.2, 142.6, 135.2, 131.2, 130.9, 130.7, 129.9, 129.0, 128.0, 127.5, 125.4, 124.6, 122.9, 121.8, 120.9, 115.1, 105.9, 100.6, 68.8, 68.6, 32.4, 32.3, 30.1, 30.1, 30.1, 30.0, 29.8, 29.8, 29.8, 29.7, 29.6, 26.5, 26.5, 23.1, 23.1, 14.3, 14.3. HRMS (MALDI) m/z calcd. for [$C_{92}H_{126}N_2O_7$], 1370.9566, found 1370.9532.

6. Absorption and emission spectra of selected photosensitizers 19

Figure S1. Absorption and emission spectra of photosensitizer **19** and its intermediates **7**, **11**, **14** and **17** dissolved in THF (0.01 M).

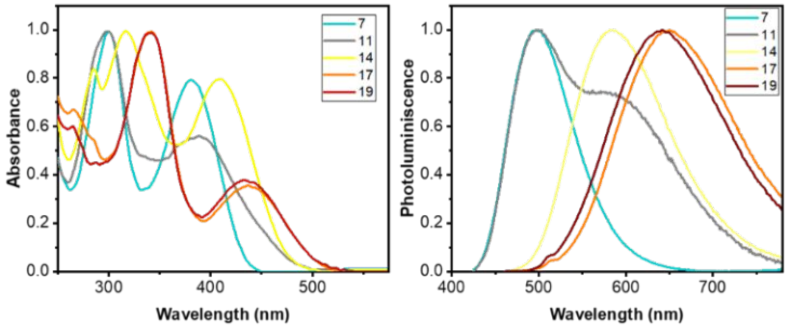

7. UV–Vis and fluorescence spectra for dyes MS5 2, 19 and 20.

Figure S2. UV–Vis (full line) and fluorescence (dashed line) spectra for dyes MS5 **2**, **19** and **20** dissolved in THF. The excitation wavelengths were 461, 434, and 427 nm, respectively.

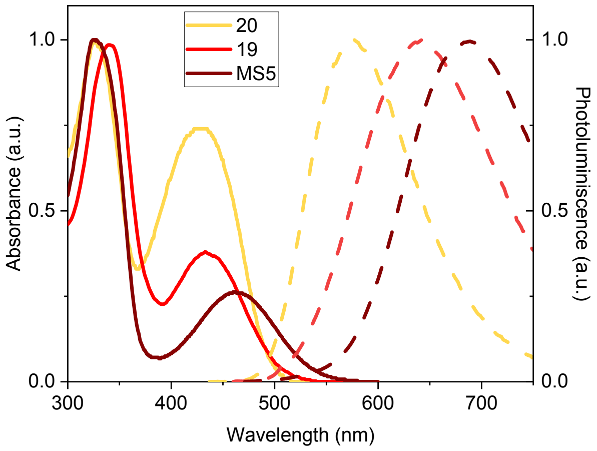

The UV-Vis spectroscopic characterization indicated that compounds **19** and **20** exhibited a maximum absorption wavelength of around 430 nm, 30 nm below the reference dye MS5 **2.** In comparison, their maximum emission wavelength was 577 nm for compound **20** and 643 nm for dye **19**, close to the emission exhibited by MS5 **2** at 684 nm.

8. Cyclic voltammogram curves of dyes MS5, 19 and 20.

Figure S3. A glassy carbon electrode was used as the working electrode, a platinum wire was used as the counter electrode, and a silver wire (Ag/Ag^+ immersed in a 0.1 M tetrabutylammonium hexafluorophosphate ($TBAPF_6$) solution in acetonitrile was used as pseudoreference electrode, calibrated with Fc^+/Fc.

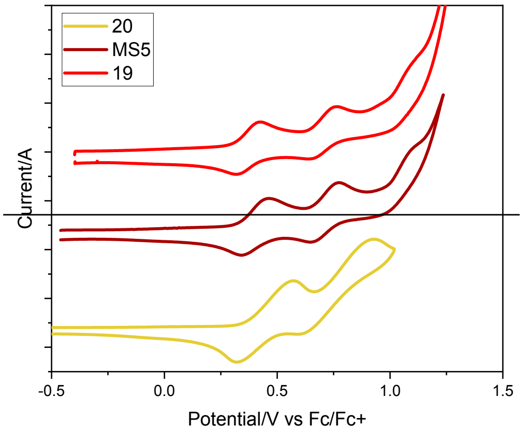

Compounds **2**, **19** and **20** presented two oxidation potentials (E_{ox}) where the first E_{ox} of **19** (0.41 V) is close to **2** (0.46 V) while **20** is more positive (0.57 V), suggesting in both cases a sufficient driving force for dye regeneration.

9. Optical and electrochemical properties of dyes MS5, 19 and 20.

Table S1. Optical and electrochemical properties of commercial dye MS5 **2** and the novel synthetic anthranil dyes **19** and **20**.

**Comp.**	**λ_{max} (abs)[a]**	**λ_{max} (em)[b]**	**E_g(eV)[c]**	**HOMO (eV)[d]**	**LUMO (eV)[e]**
**19**	434	643	2.46	-5,17	-2.72
**20**	428	577	2.52	-5.25	-2.73
MS5 **2**	463	684	2.29	-5.20	-2.87

[a] Measured in THF solution at a 10−5 mol L−1 concentration. [b] Emission measured at 434 nm (**19**), 427 nm (**20**) and 461 nm (**2**). [c] Estimated from the absorption edge in solution using the equation $E_g = 1240/\lambda_{onset}$ (eV). [d] The HOMO energy level was obtained in ACN with ferrocene as an external reference. [e]Calculated by E_g+E_{HOMO}.

The narrower energy gap observed for the anthranilic dye **19** (2.46 eV) indicates that in this molecule, the acceptor group can more efficiently extract the charge density from the donor group than its analogue **20** (2.52 eV). In addition, the exhibited energy levels ensure enough driving force for dye regeneration and electron injection into the TiO_2 conduction band within a DSSC.

10. Time-Correlated Single Photon Counting of photosensitizers in solution

Figure S4 Photoemission decay with time measured by TCSPC of the samples in THF/EtOH (1:19) solution recorded after 5000 acquisition count per second. The instrument response function is plotted in black for comparison purposes.

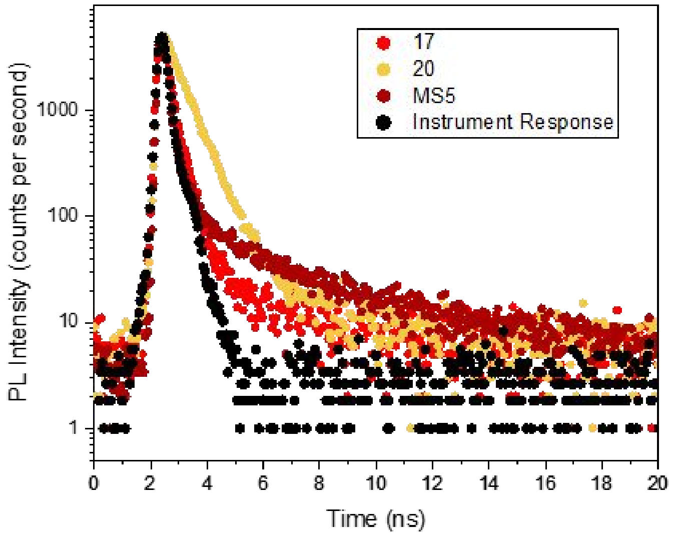

Unfortunately, the decay of the photoluminescent lifetimes resulted in being close to the instrument response (390 ps), which has prevented further studies by this technique at the dye/TiO_2 interface.

Table S2. Decay fitting parameters obtained from the measurements plotted in Figure S1, where A represents the contribution of each process and τ the lifetime.

Dye	τ_1 (ns)	A_1 (%)	τ_2 (ns)	A_2 (%)
19	0.29	94.2	3.09	5.8
**20**	0.65	96.4	4	3.6
MS5 **2**	1.18	51.2	5.6	48.8

11. TAS measurements on 4 µm transparent TiO_2 sensitized films

Preparation of transparent TiO_2 electrodes. The TiO_2 layer was prepared by screen printing achieving a total thickness of approximately 4 µm. The electrodes were sensitized in 0.1 mM solutions of the respective dye in THF/EtOH (1:19).

Figure S5. Transient absorption spectra of MS5 **2**, **19** and **20** at 3 µs after excitation with a 420 nm laser.

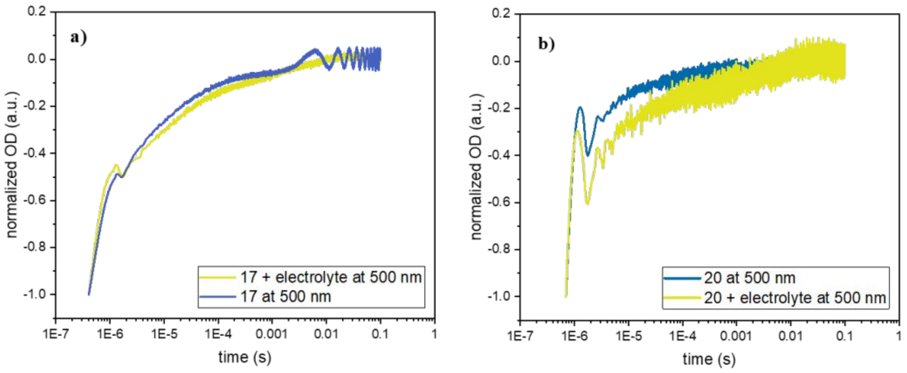

After excitation at 420 nm, all three samples presented the ground state bleaching around 500 nm, followed by the excited state absorption of the dye detected at 600 and 920 nm.

Figure S6. Comparison of the transient absorption decays of a) **19** and b) **20** titania sensitized films in contact with the copper electrolyte recorded at 920 and 500 nm after excitation at 420 nm.

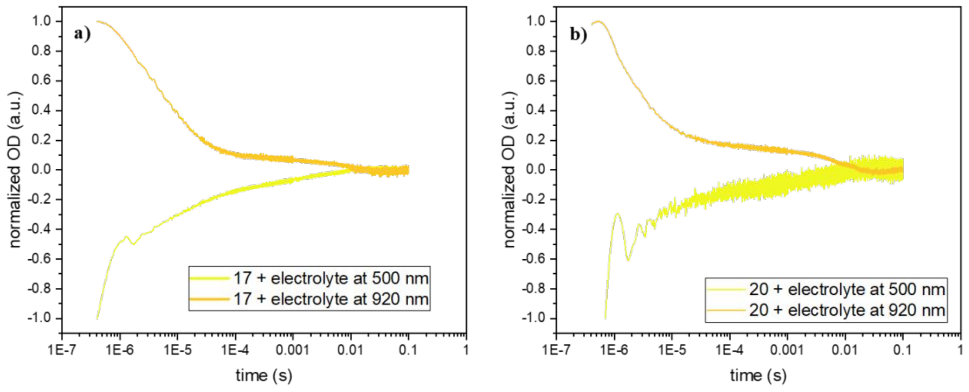

12. Transient Absorption Spectra (TAS) experiments

Figure S7. Transient absorption spectra of MS5 **2**, **19** and **20** at 3 μs after excitation with a 420 nm laser.

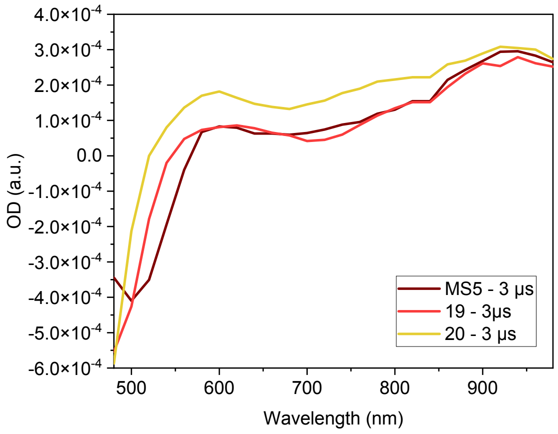

13. Transient absorption decays for dyes MS5, 19 and 20

Figure S8. Transient absorption decays for dye-sensitized TiO_2 films without or with Cu^{I}/Cu^{II} based electrolyte recorded at 920 nm. λ_{exc} 420 nm.

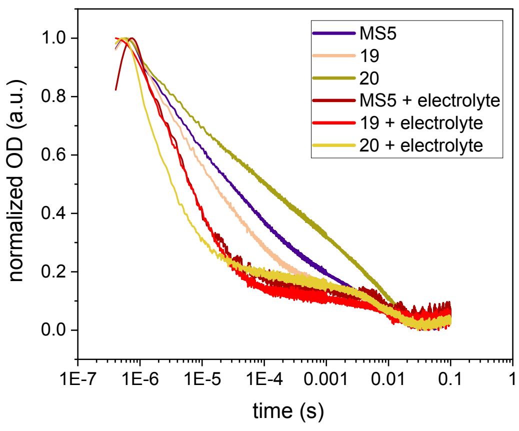

A faster signal decay is appreciated in the three samples when the electrolyte is added, revealing the regeneration efficiency of the electrolyte. This is especially evident for dye **20**, which shows the longest-lived excited state in dye/TiO_2 but the fastest decay in the presence of the electrolyte

14. ^{1}H and ^{13}C NMR Spectra

Figure S9. ^{1}H-NMR and ^{13}C {^{1}H} NMR spectra of 7-bromobenzo[*c*]isoxazole **5**:

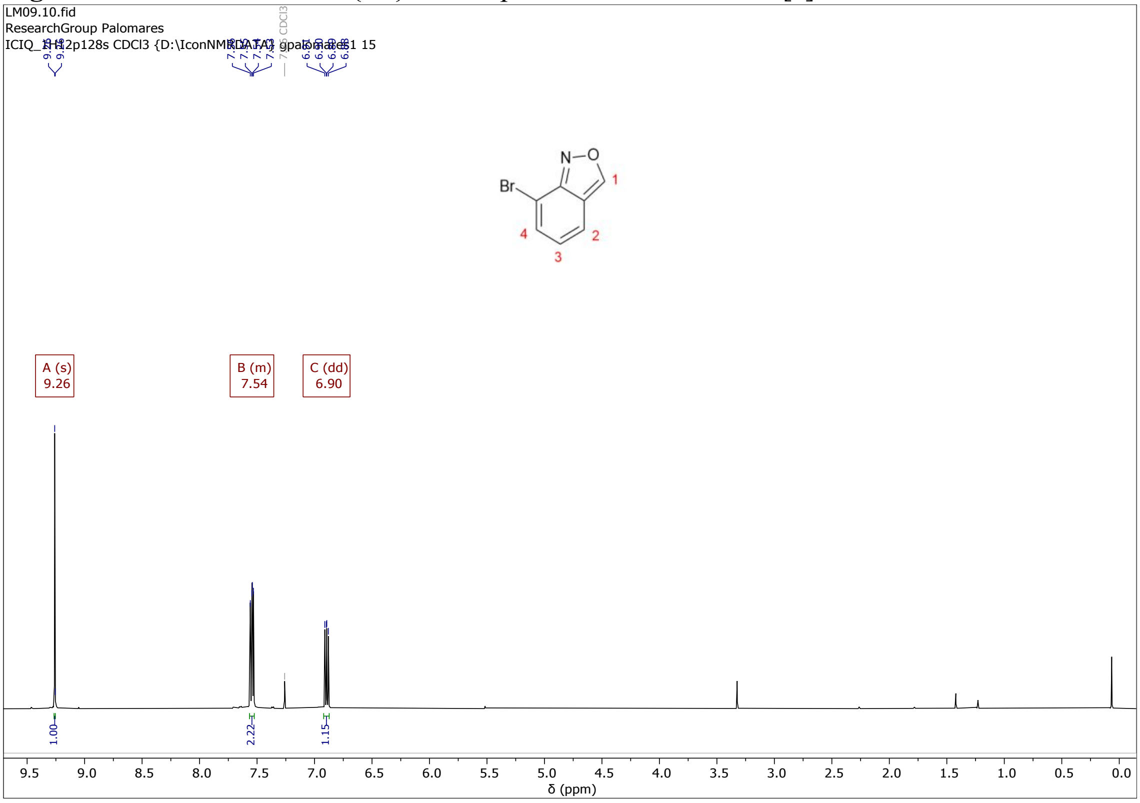

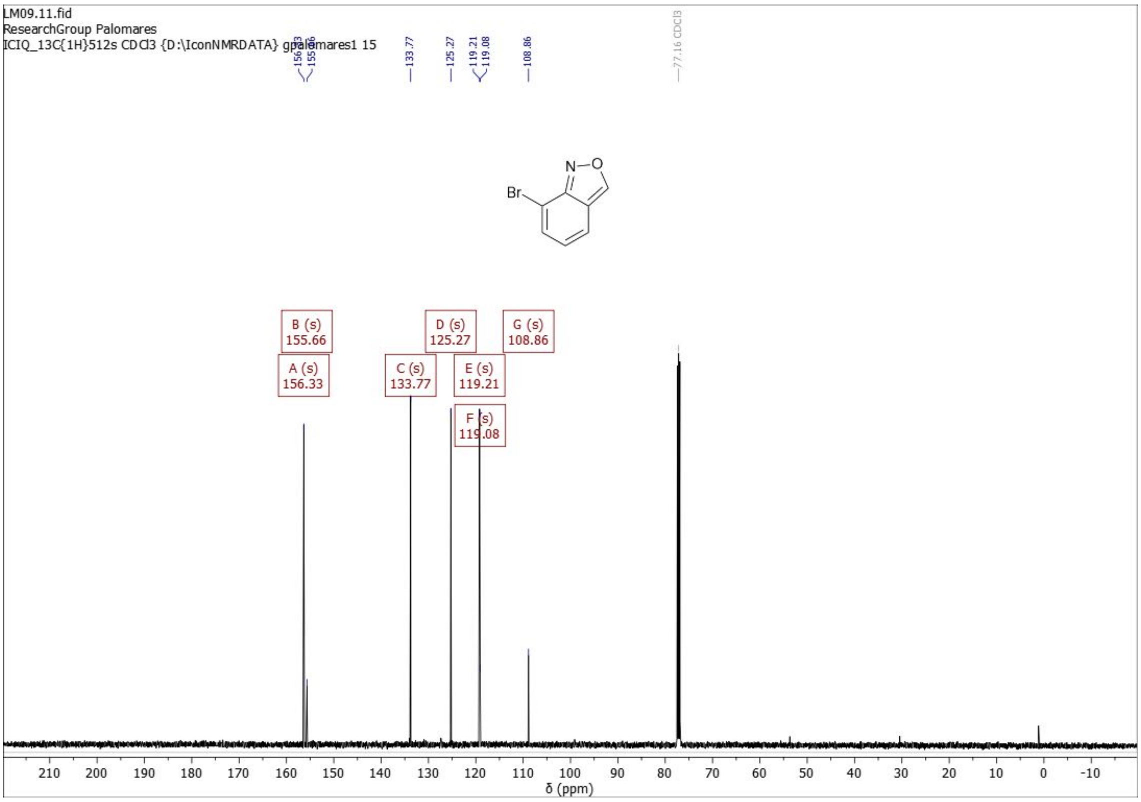

Figure S10. ^{1}H-NMR and ^{13}C {^{1}H} NMR spectra of *N,N*-diphenyl-4-(4,4,5,5-tetramethyl-1,3,2-dioxoborolan-2-yl)aniline **6**:

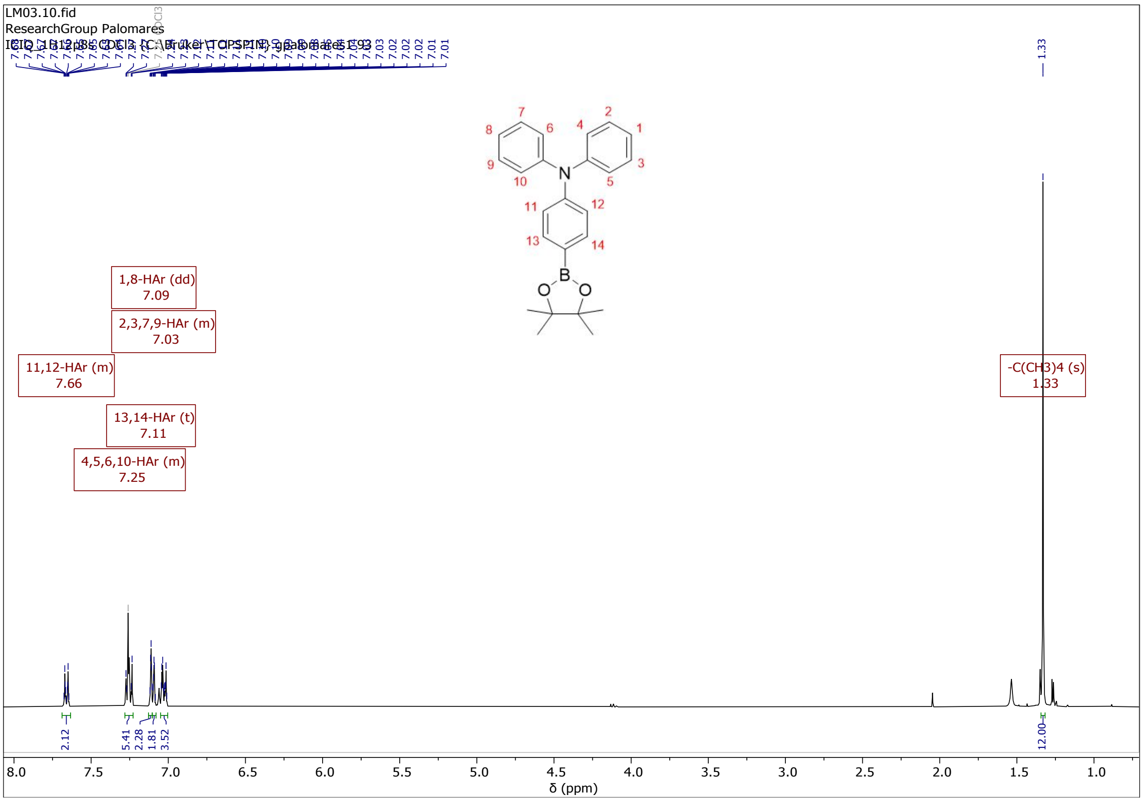

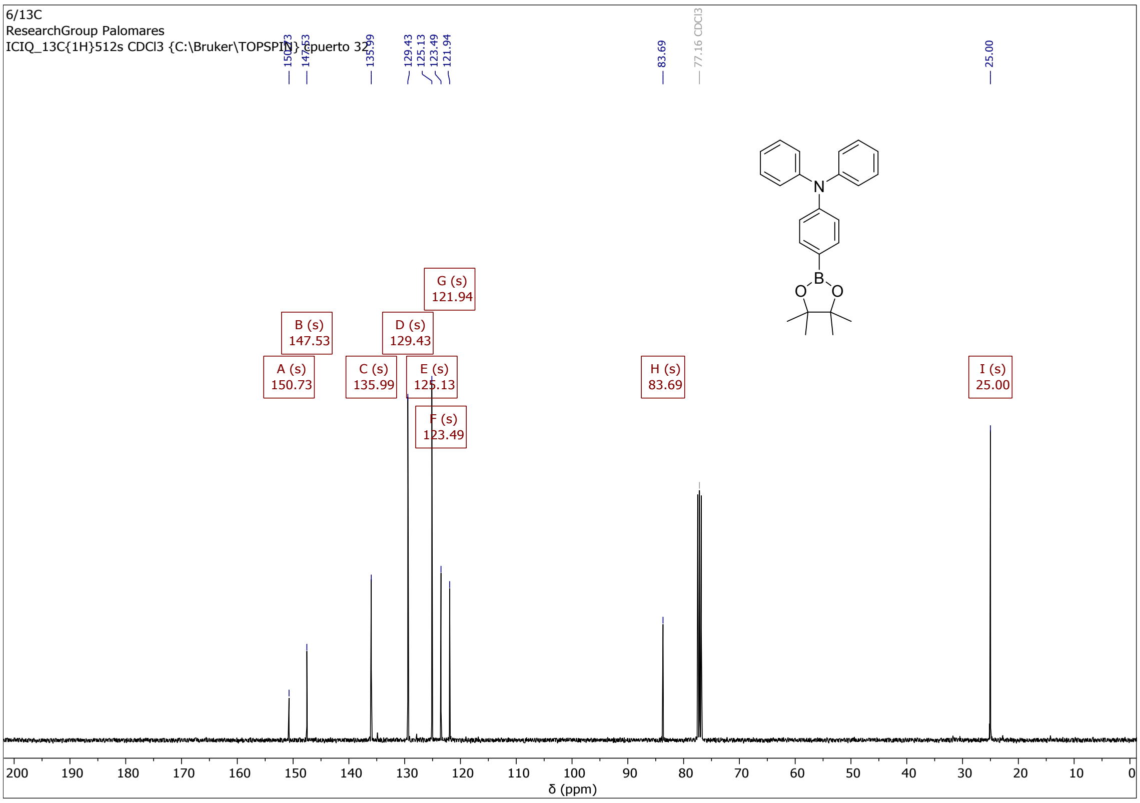

Figure S11. ^{1}H-NMR and ^{13}C {^{1}H} NMR spectra of 1-bromo-2,4-bis(dodecycloxy)benzene **SI1**:

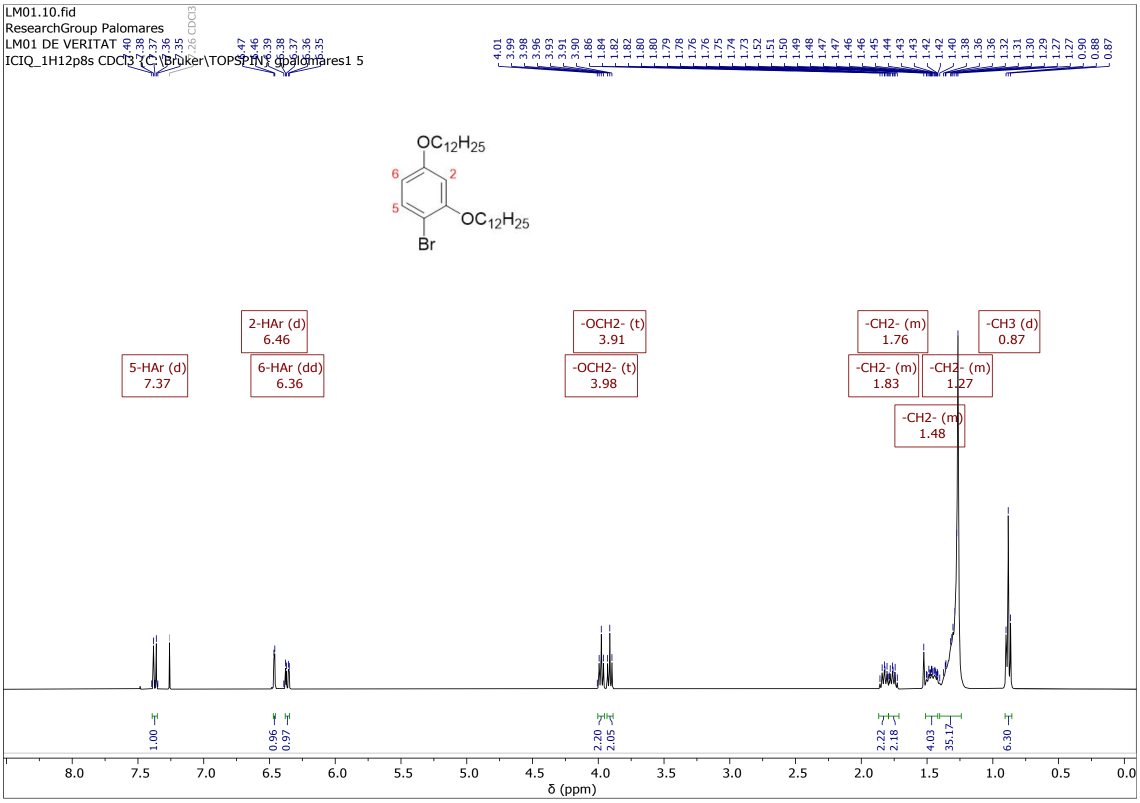

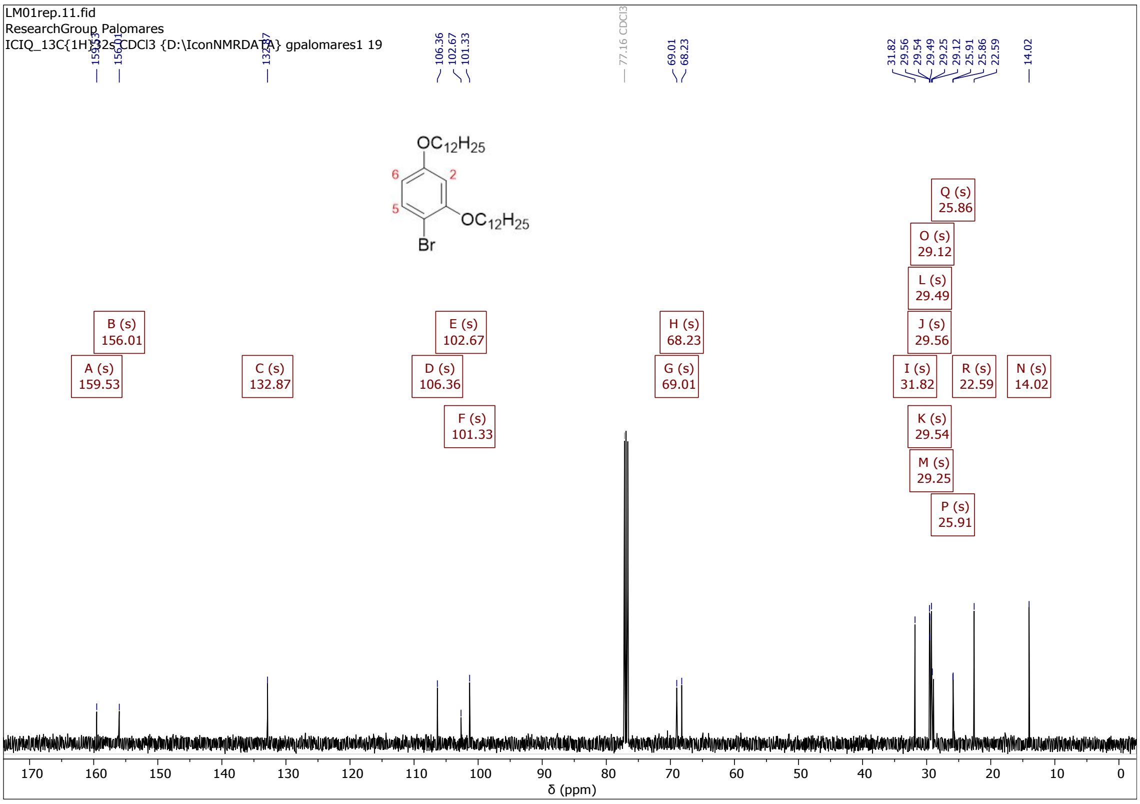

Figure S12. ^{1}H-NMR and ^{13}C {^{1}H} NMR spectra of 2-(2,4-bis(dodecyloxy)phenyl)-4,4,5,5-tetramethyl-1,3,2-dioxaborolane **16**:

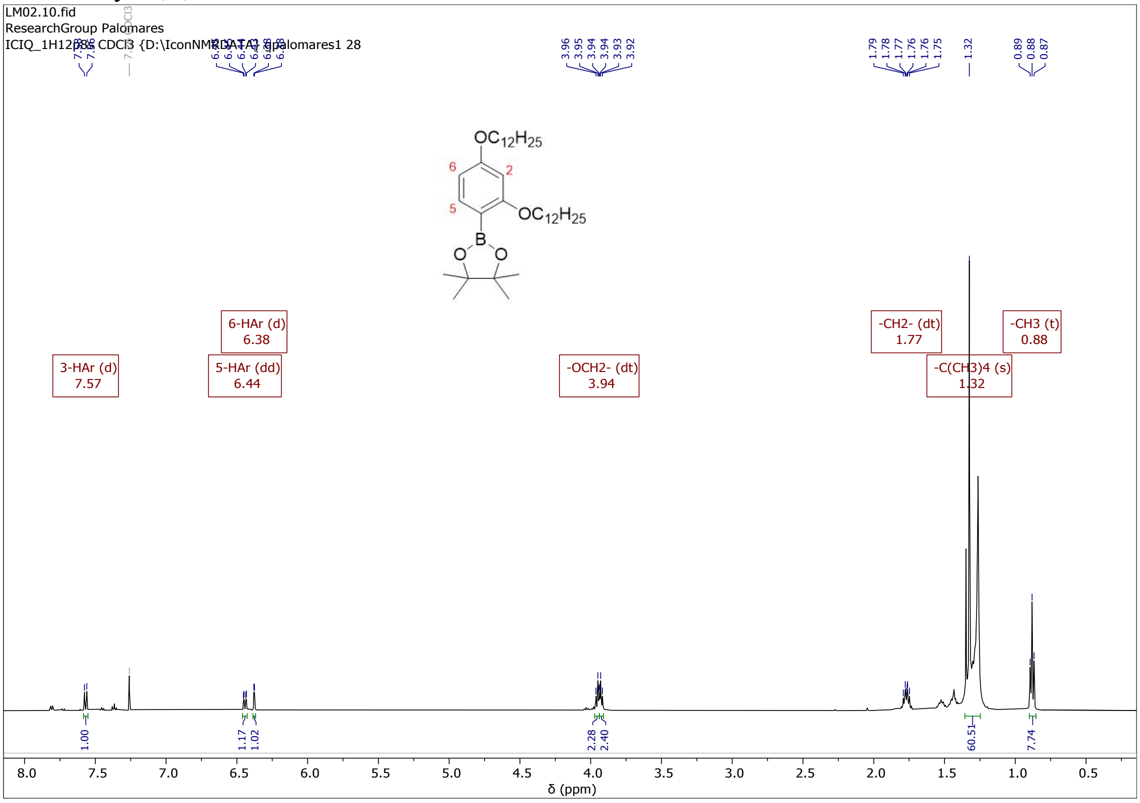

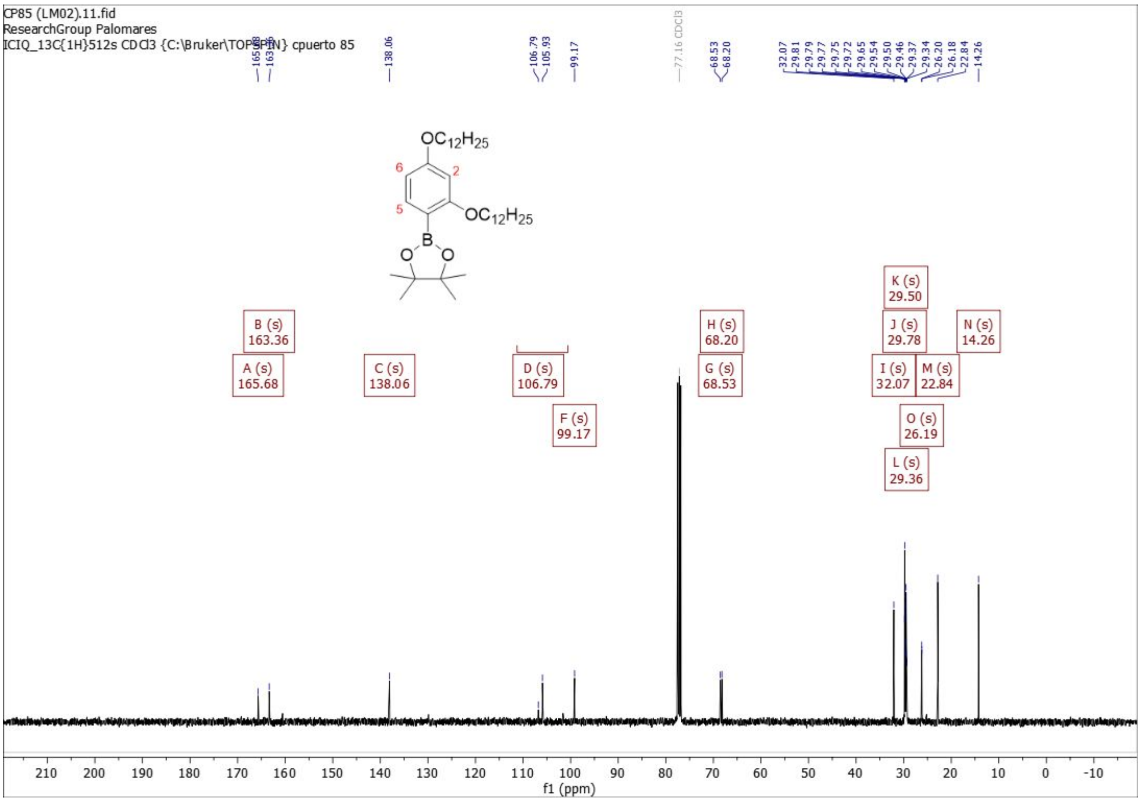

Figure S13. ^{1}H-NMR and ^{13}C {^{1}H} NMR spectra of 4-(benzo[*c*]isoxazol-7-yl)-*N,N*-diphenylaniline **7**:

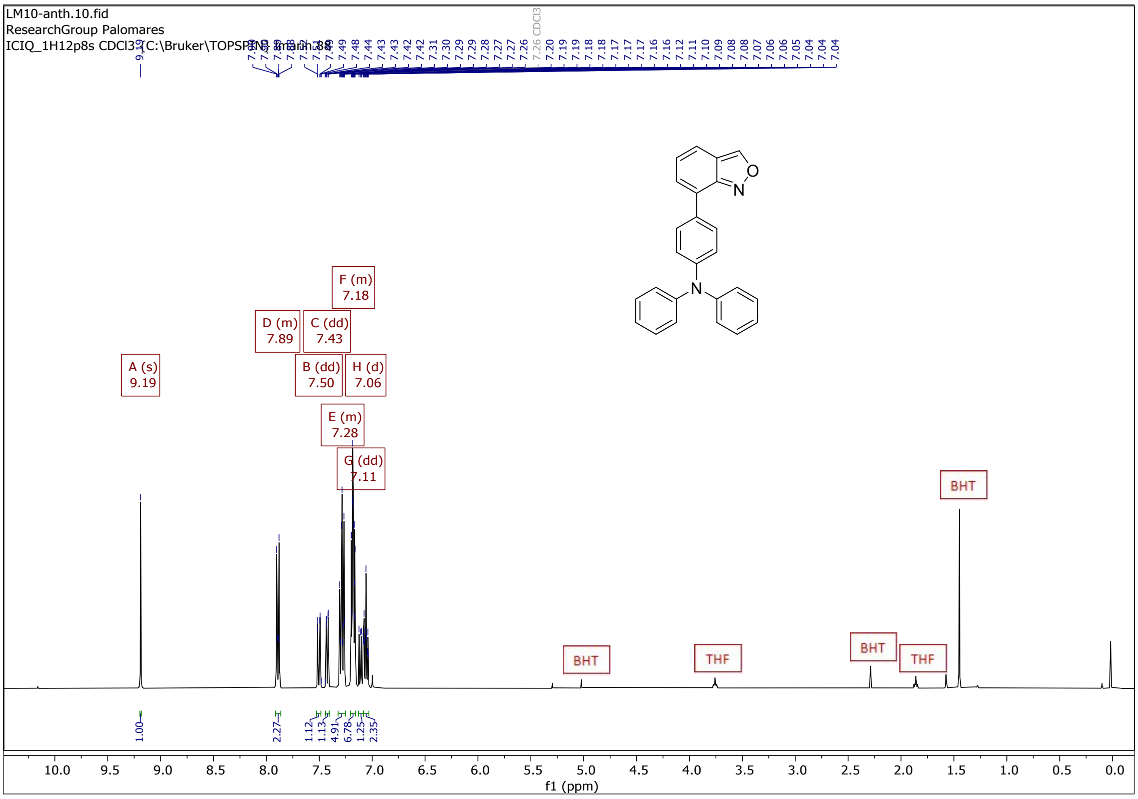

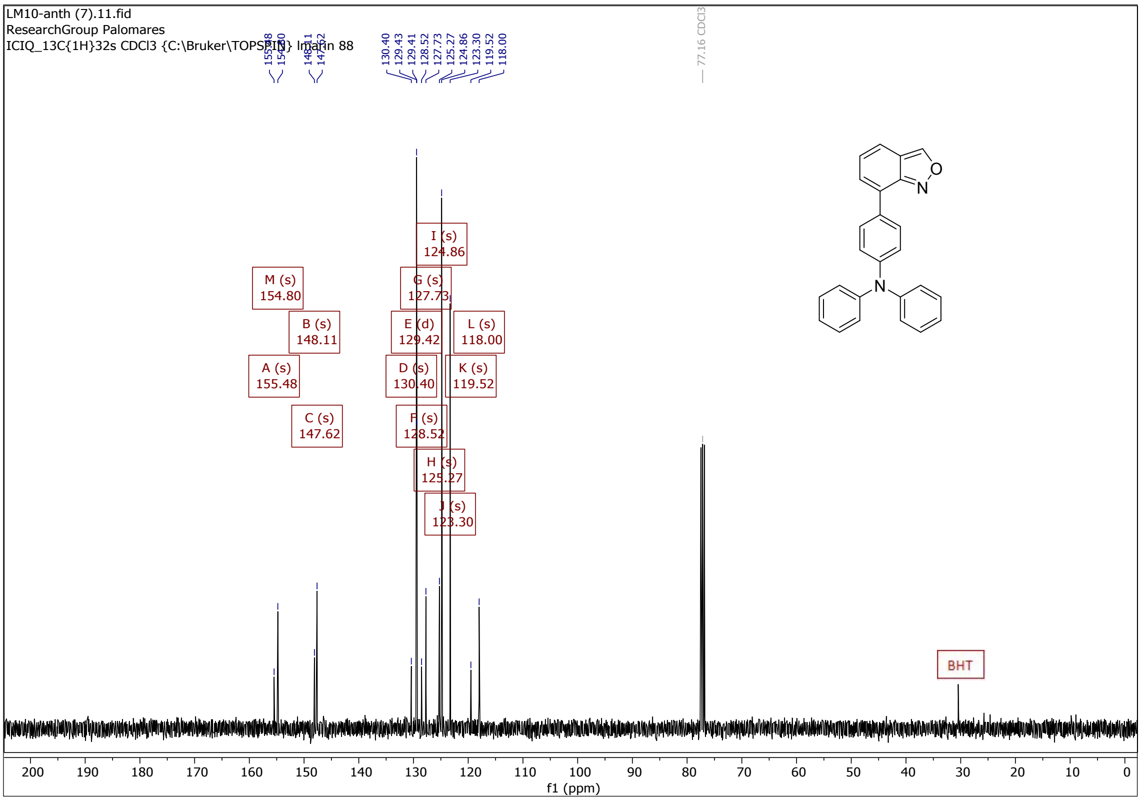

Figure S14. ^{1}H-NMR and ^{13}C {^{1}H} NMR spectra of ethyl 4-(7-(4-(diphenylamino)phenyl)benzo[*c*] isoxazol-3-yl)benzoate **11**:

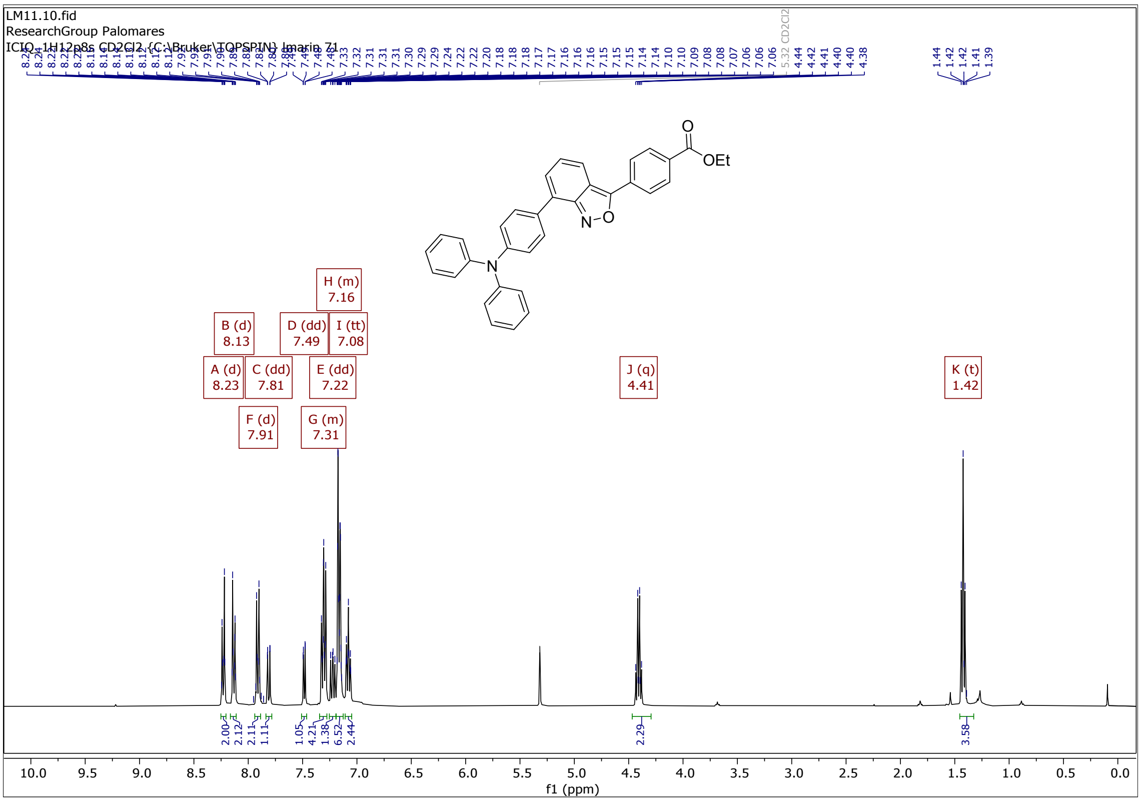

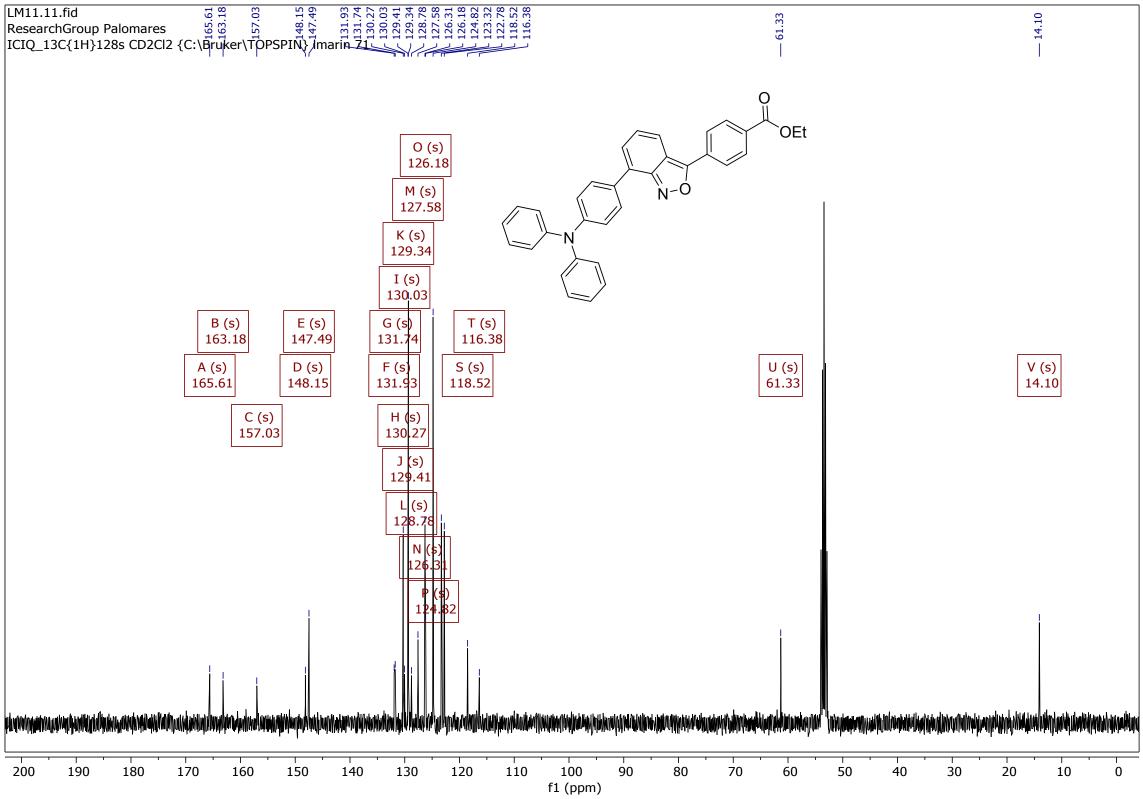

Figure S15. ^{1}H-NMR and ^{13}C {^{1}H} NMR spectra of ethyl 4-(7-(4-(bis(4-bromophenyl)amino)phenyl)benzo[*c*]isoxazol-3-yl)benzoate **14**:

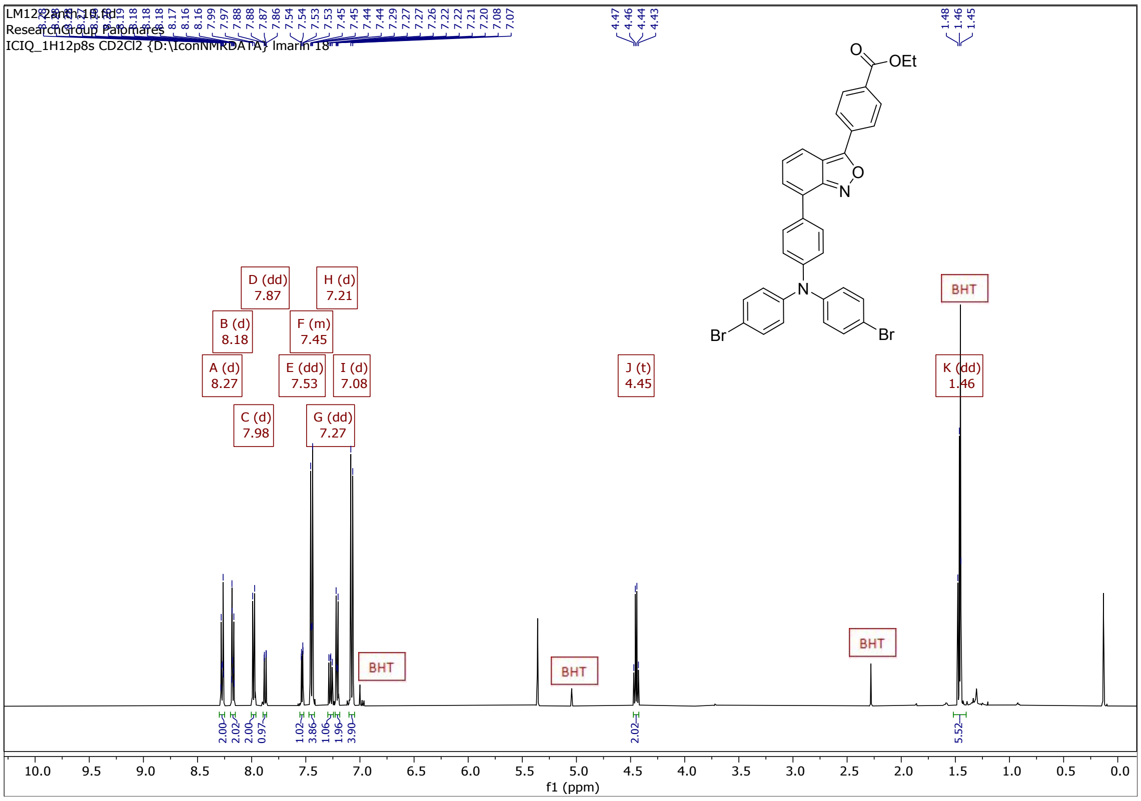

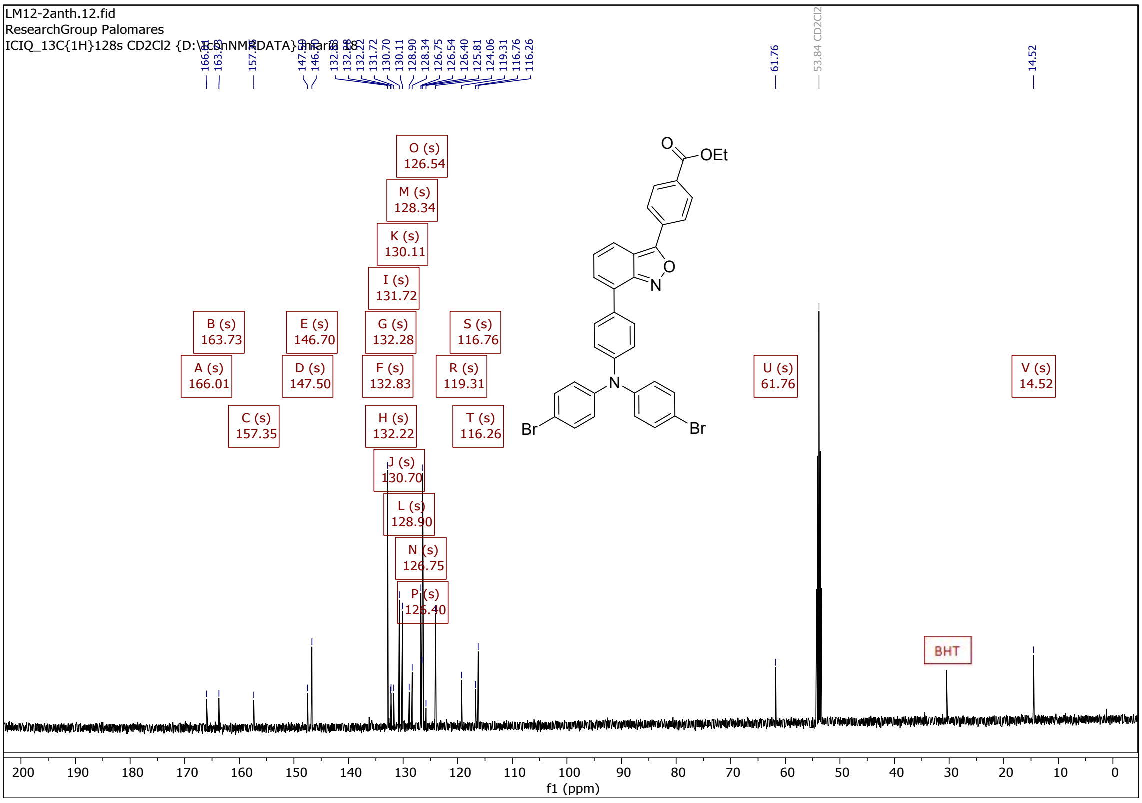

Figure S16. ^{1}H-NMR and ^{13}C {^{1}H} NMR spectra of ethyl 4-(7-(4-(bis(2',4'-bis(dodecycloxy)-[1,1'-biphenyl]-4-yl)amino) phenyl)benzo[*c*]isoxazole-3-yl)benzoate **17**:

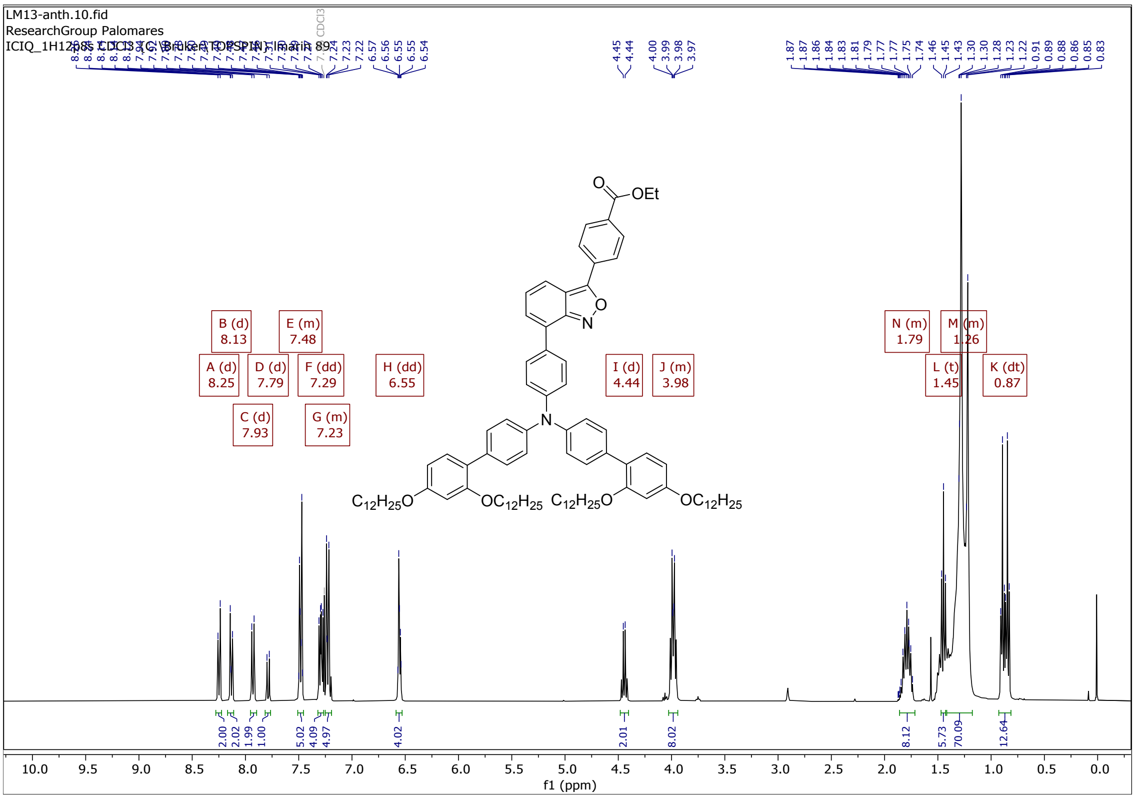

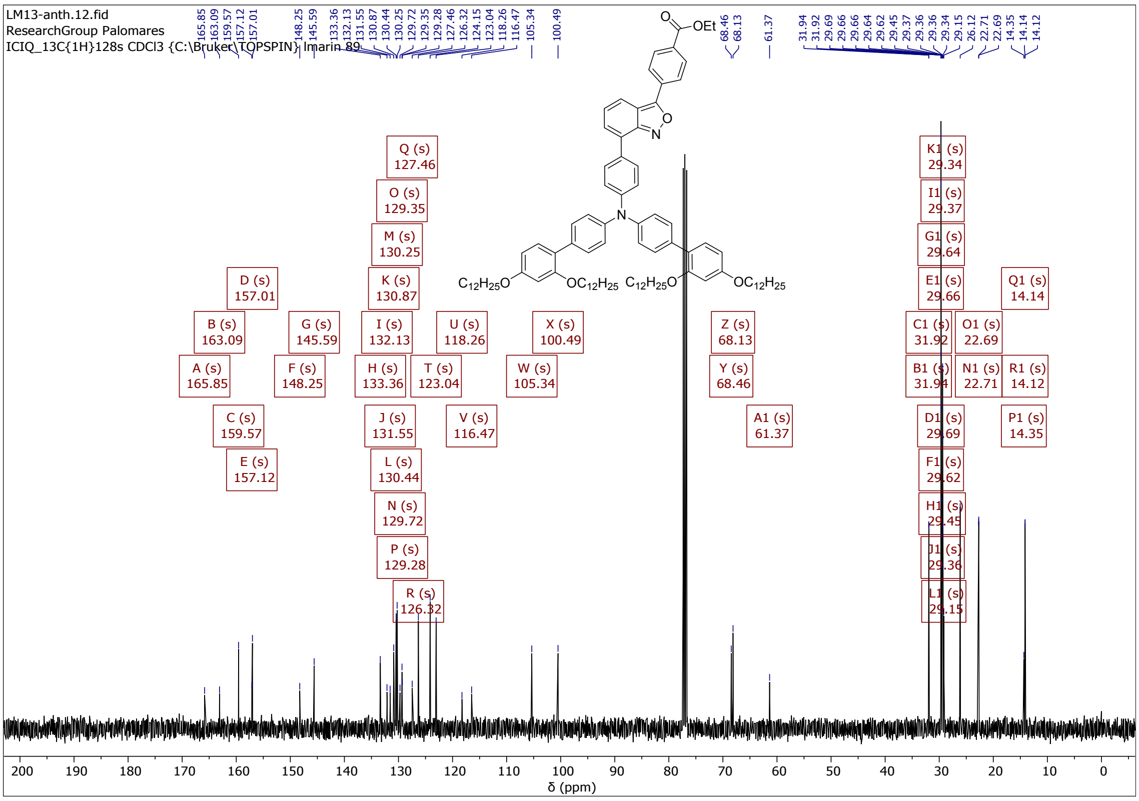

Figure S17. 1D- and 2D-NMR spectra of 4-(7-(4-(bis(2',4'-bis(dodecycloxy)-[1,1'-biphenyl]-4-yl)amino)phenyl)benzo[*c*]isoxazole-3-yl)benzoic acid **19**:

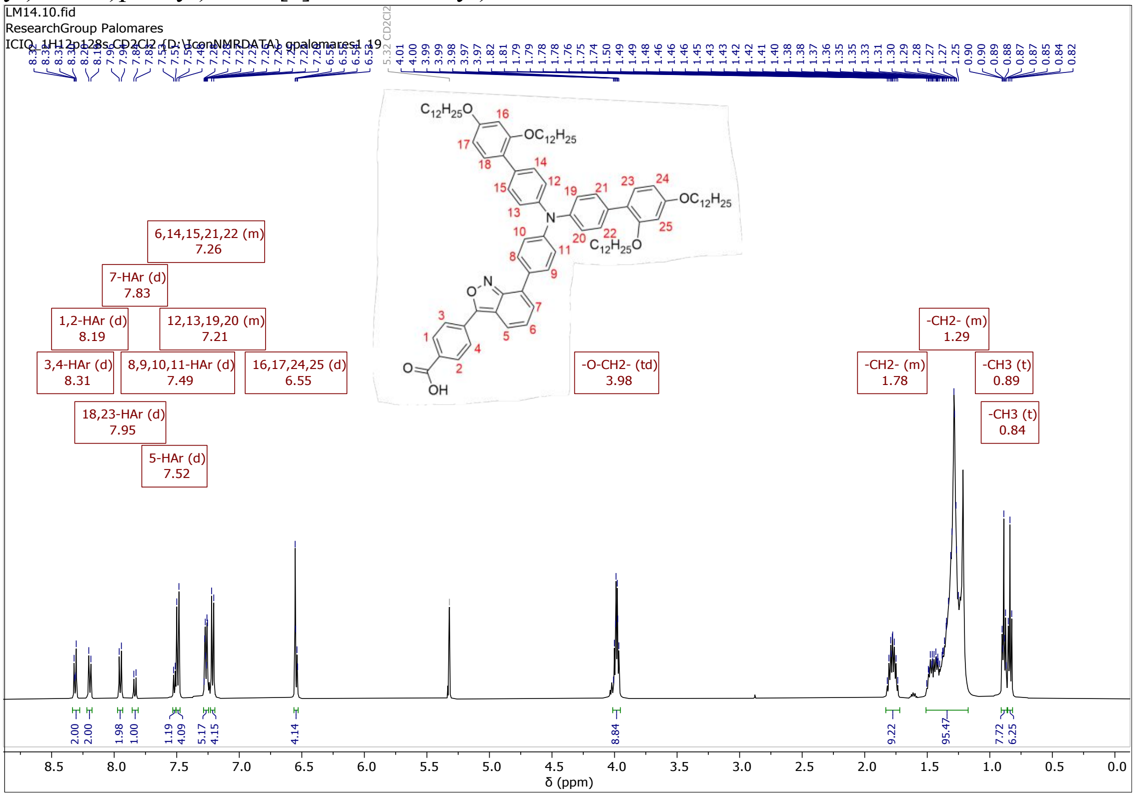

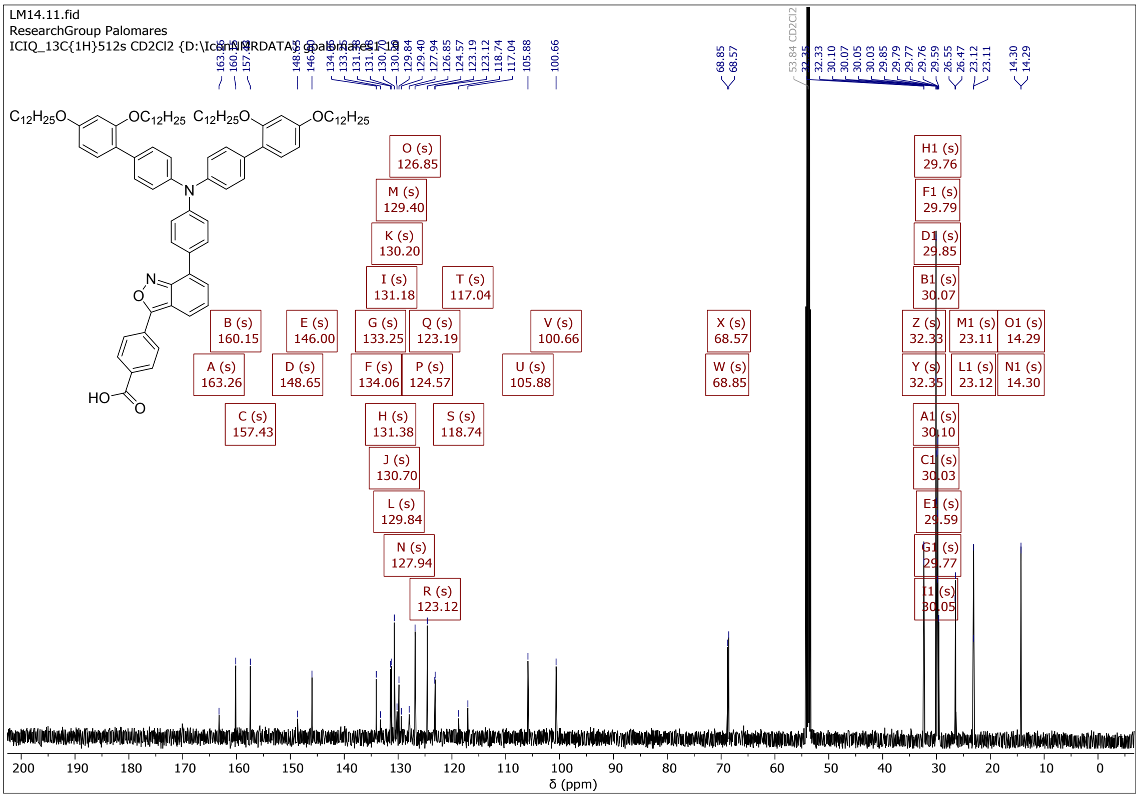

LM14.11.fid
ResearchGroup Palomares
ICIQ_13C{1H}512s CD2Cl2 {D:\IconNMRDATA} gpalomares 1 19
A (s) 163.26
B (s) 160.15
C (s) 157.43
D (s) 148.65
E (s) 146.00
F (s) 134.06
G (s) 133.25
H (s) 131.38
I (s) 131.18
J (s) 130.70
K (s) 130.20
L (s) 129.84
M (s) 129.40
N (s) 127.94
O (s) 126.85
P (s) 124.57
Q (s) 123.19
R (s) 123.12
S (s) 118.74
T (s) 117.04
U (s) 105.88
V (s) 100.66
W (s) 68.85
X (s) 68.57
Y (s) 32.35
Z (s) 32.33
A1 (s) 30.10
B1 (s) 30.07
C1 (s) 30.03
D1 (s) 29.85
E1 (s) 29.59
F1 (s) 29.79
G1 (s) 29.77
H1 (s) 29.76
I1 (s) 30.05
L1 (s) 23.12
M1 (s) 23.11
N1 (s) 14.30
O1 (s) 14.29
53.84 CD2Cl2
δ (ppm)

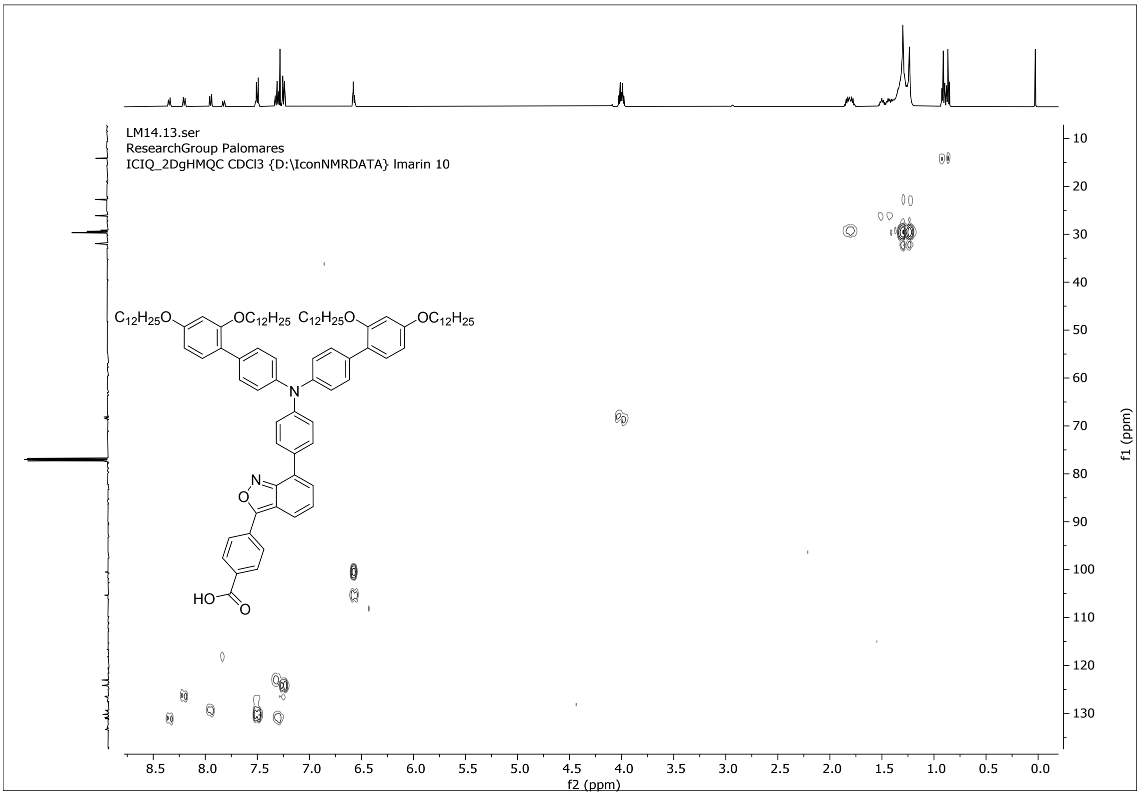

LM14.13.ser
ResearchGroup Palomares
ICIQ_2DgHMQC CDCl3 {D:\IconNMRDATA} lmarin 10
f2 (ppm)
f1 (ppm)

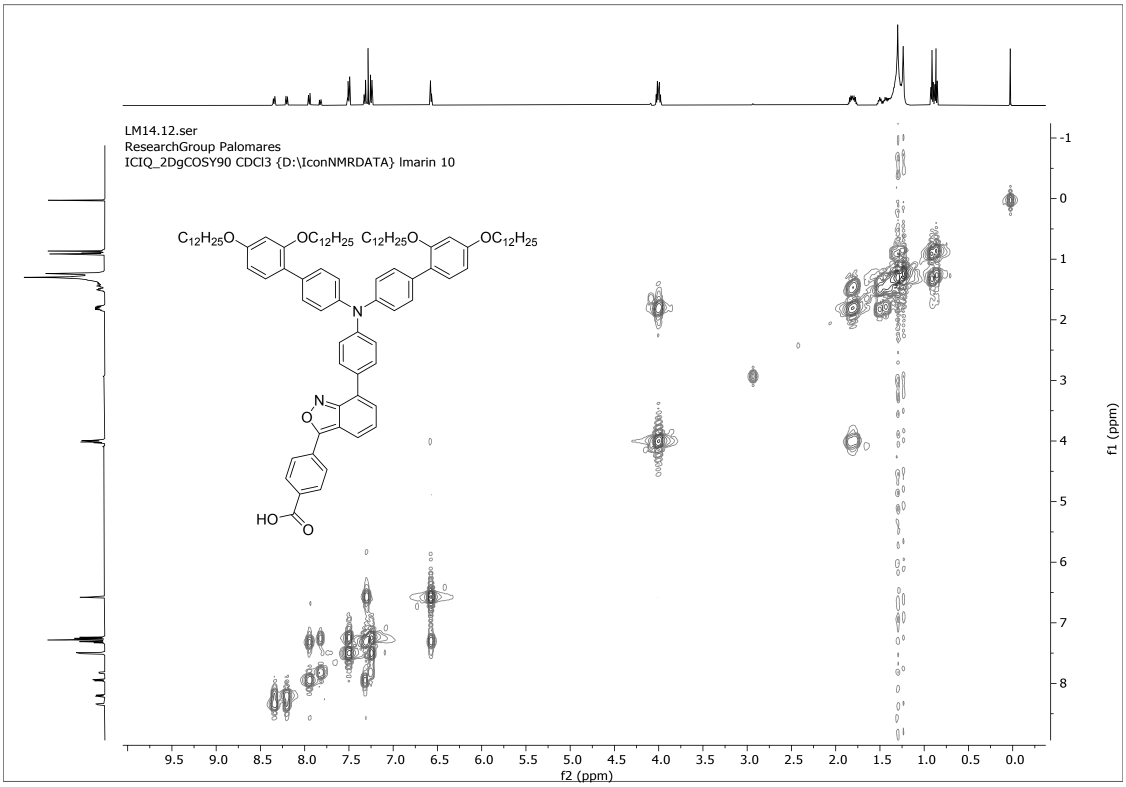
LM14.12.ser
ResearchGroup Palomares
ICIQ_2DgCOSY90 CDCl3 {D:\IconNMRDATA} lmarin 10
f2 (ppm)
f1 (ppm)

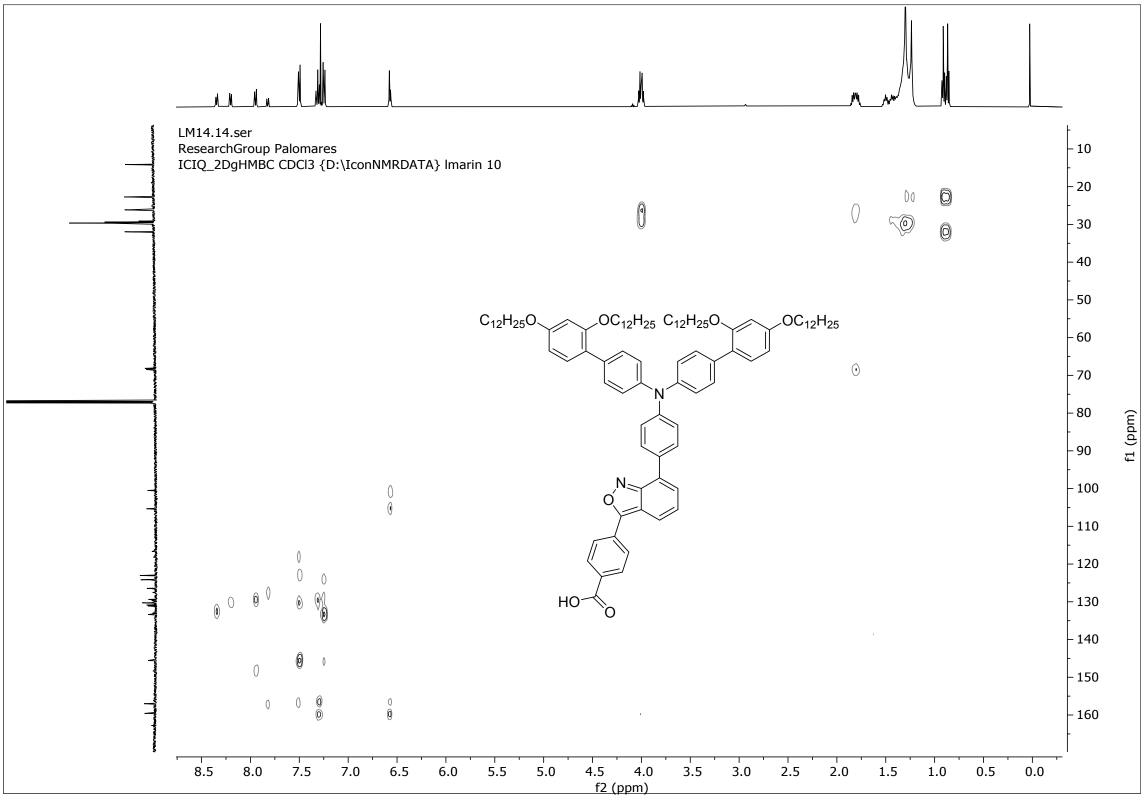
LM14.14.ser
ResearchGroup Palomares
ICIQ_2DgHMBC CDCl3 {D:\IconNMRDATA} lmarin 10
f2 (ppm)
f1 (ppm)

Figure S18. ^{1}H-NMR and ^{13}C {^{1}H} NMR spectra of ethyl 4-(benzo[*c*]isoxazol-7-yl)benzoate **9**:

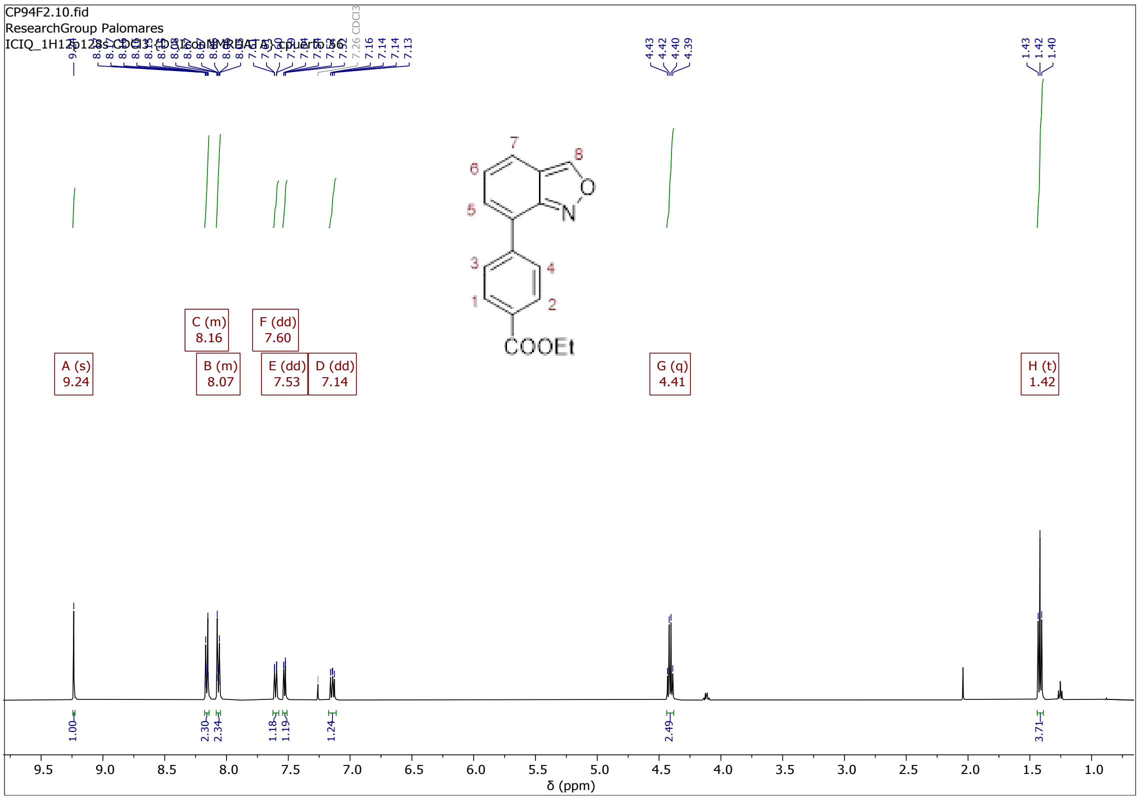

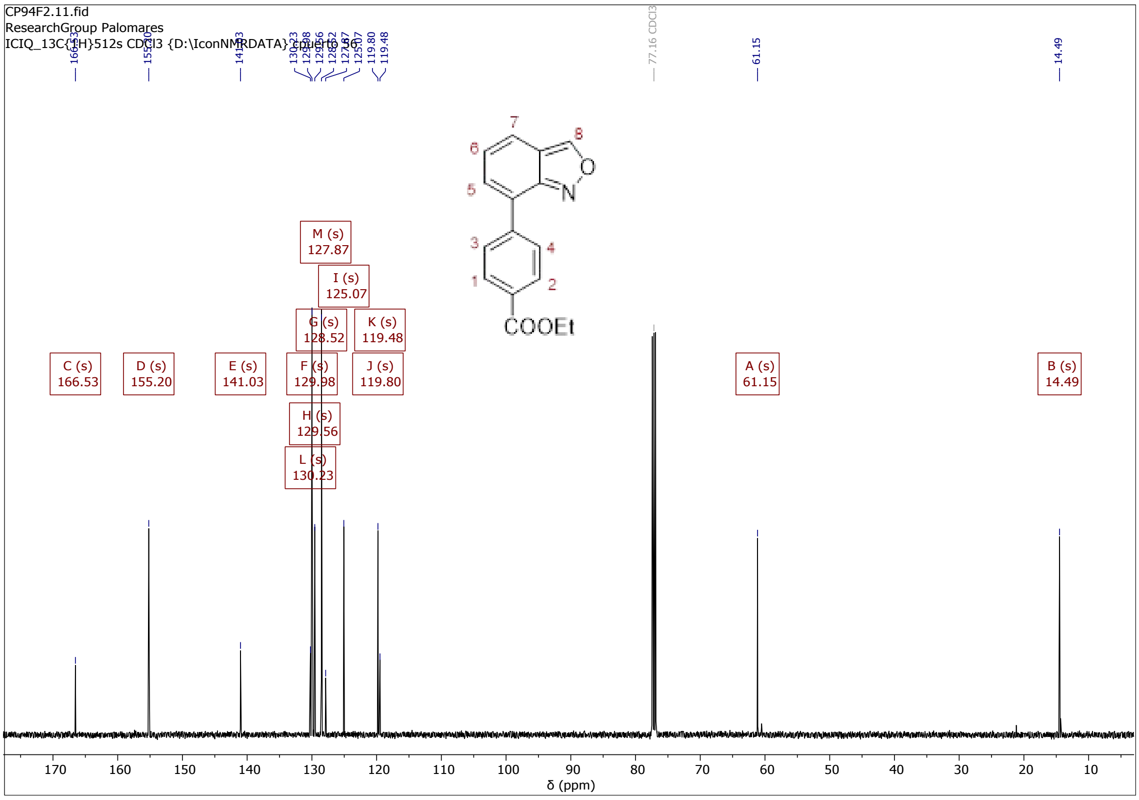

Figure S19. ^{1}H-NMR and ^{13}C {^{1}H} NMR spectra of ethyl 4-(3-(4-(diphenylamino)phenyl)benzo [*c*]isoxazol-7-yl)benzoate **13**:

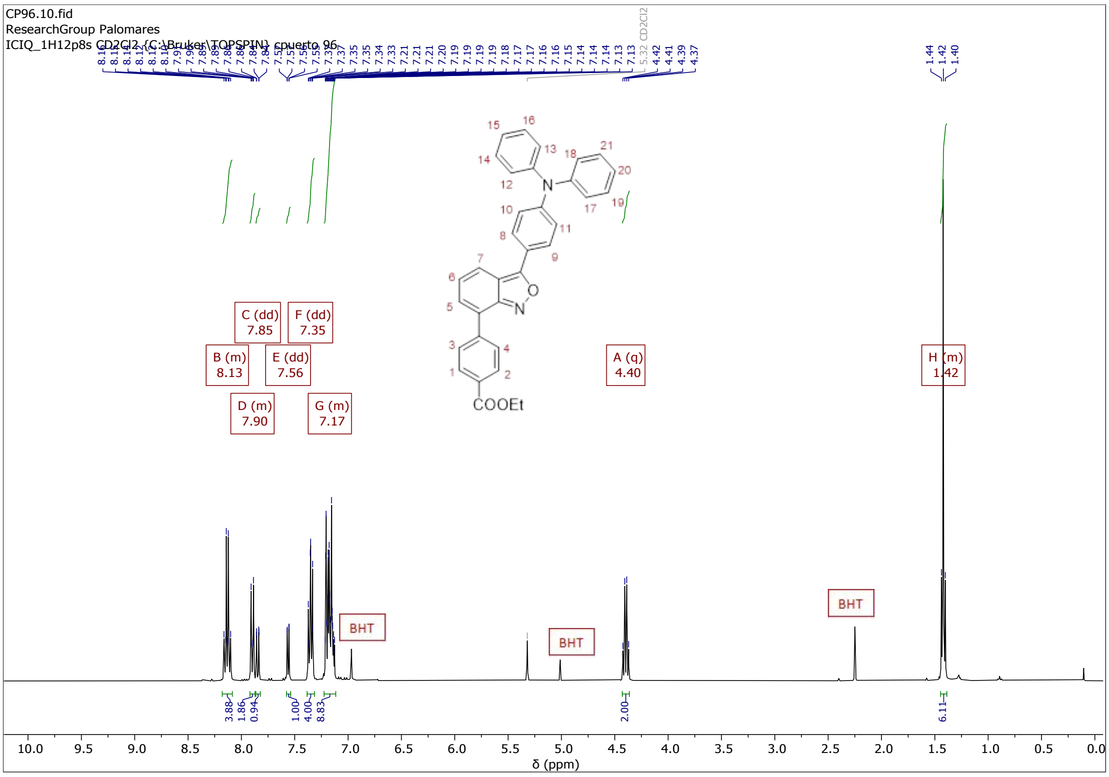

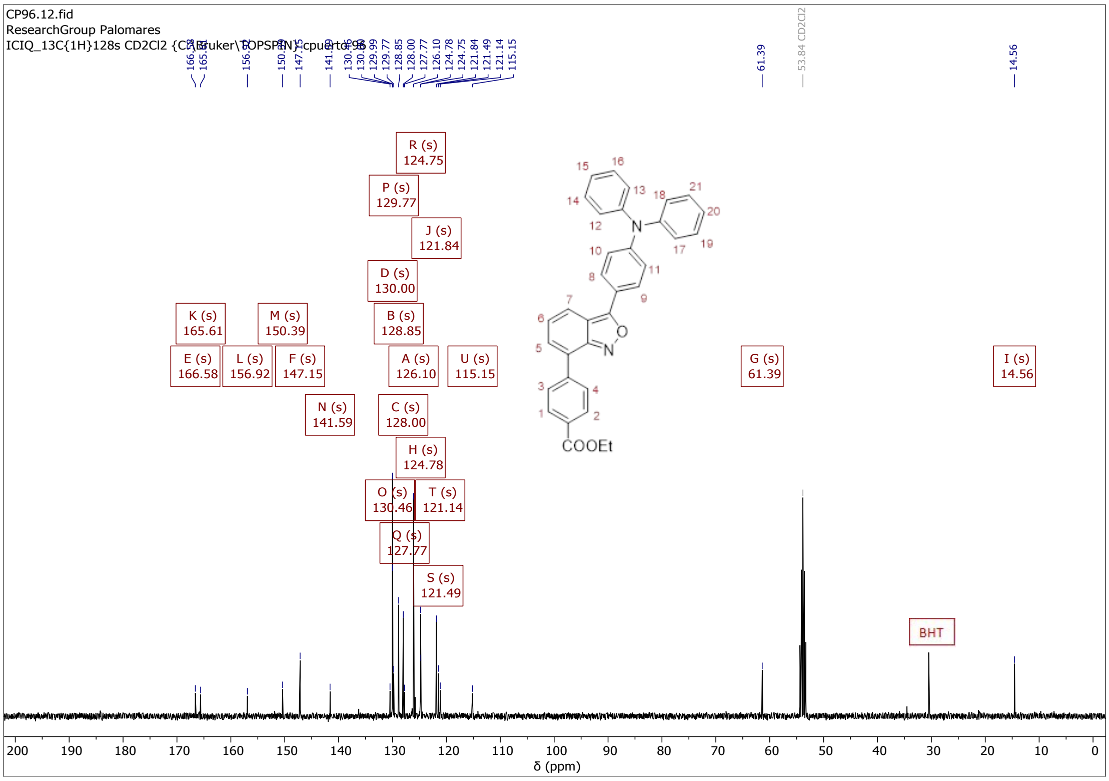

Figure S20. ^{1}H-NMR and ^{13}C {^{1}H} NMR spectra for side product **13'**.

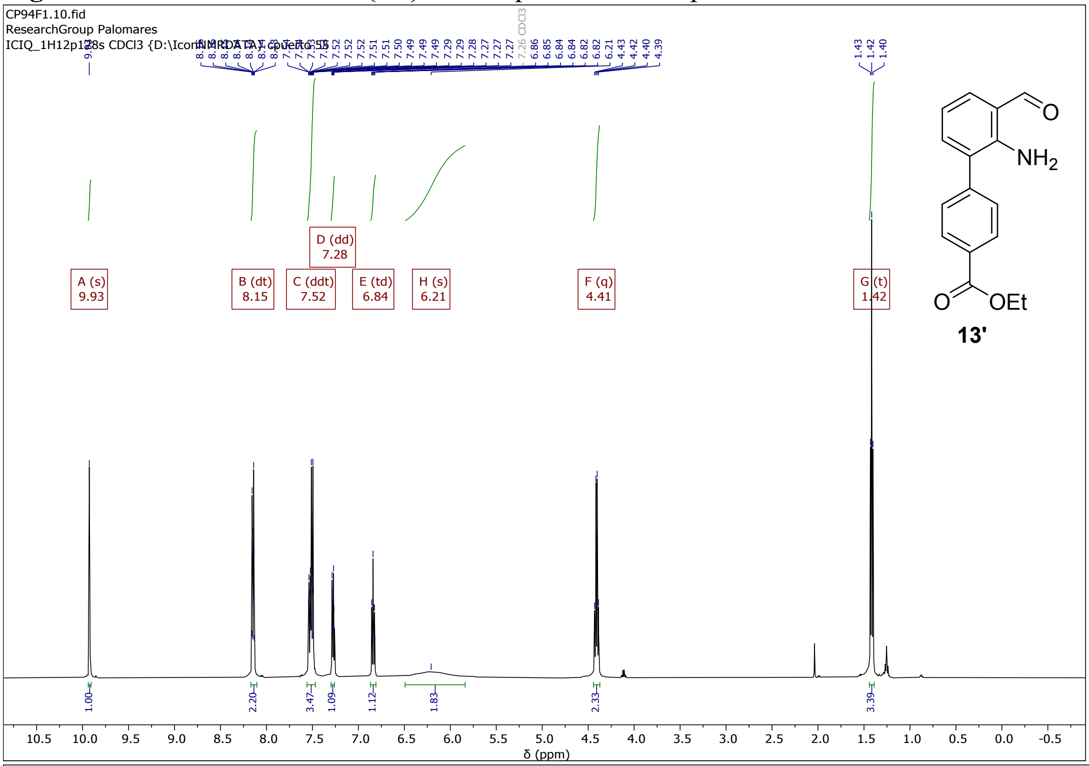

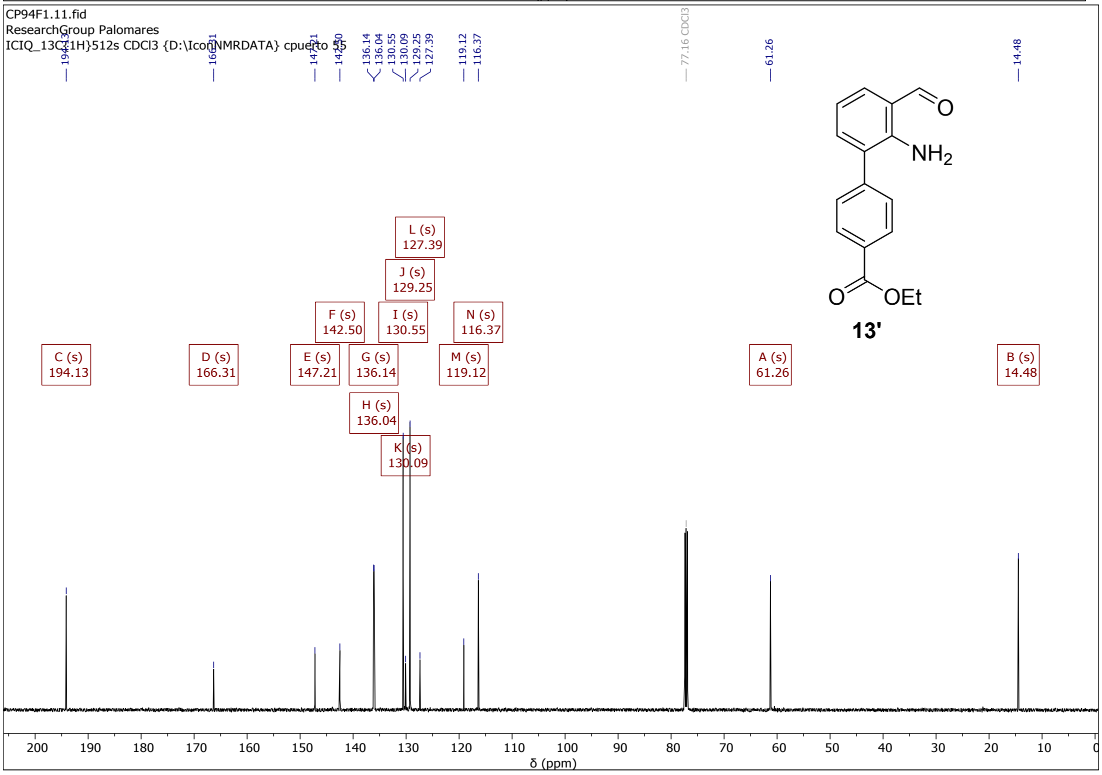

Figure S21. ^{1}H-NMR and ^{13}C {^{1}H} NMR spectra of ethyl 4-(3-(4-(bis(4-bromophenyl)amino) phenyl)benzo[*c*]isoxazol-7-yl)benzoate **15**:

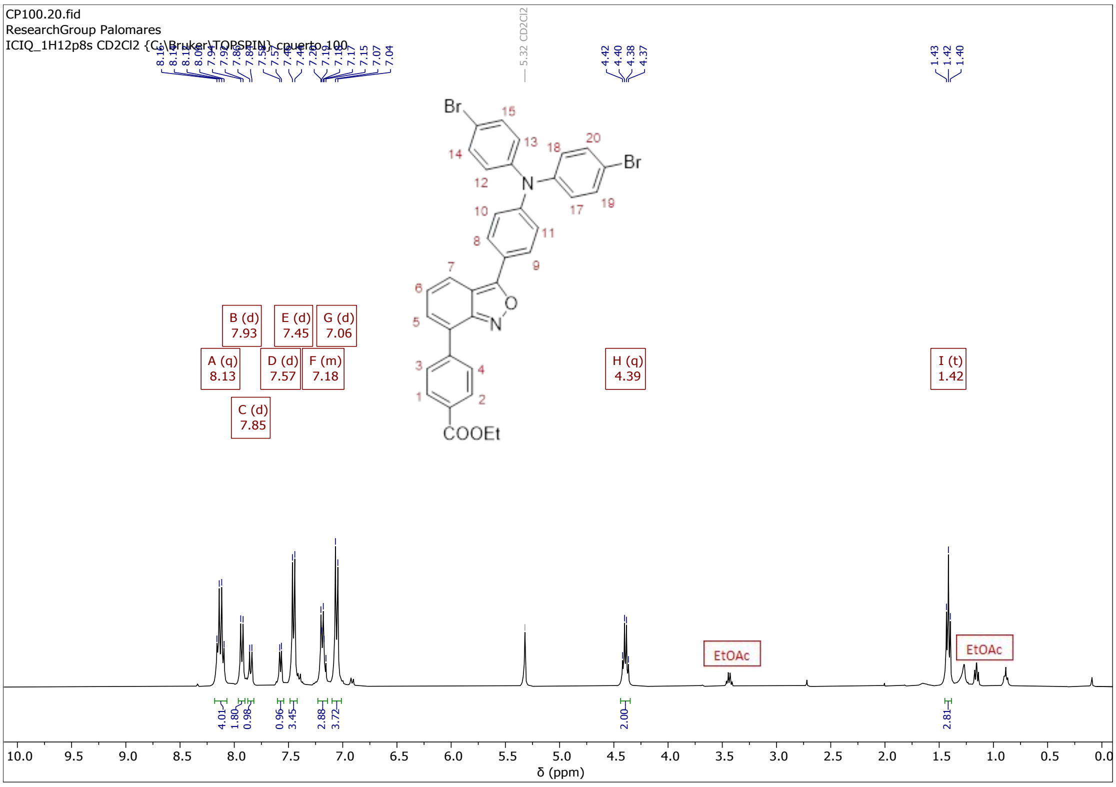

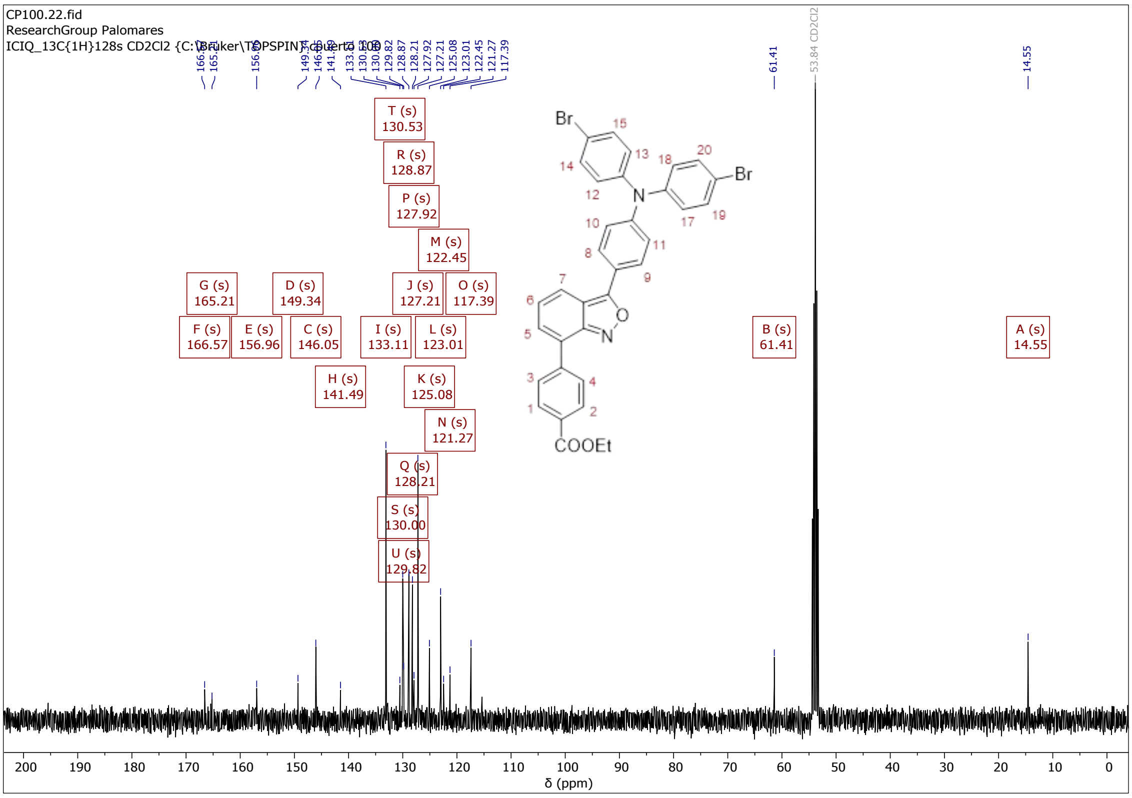

Figure S22. ^{1}H-NMR and ^{13}C {^{1}H} NMR spectra of ethyl 4-(3-(4-(bis(2',4'-bis(dodecyloxy)-[1,1'-biphenyl]-4-yl)amino)phenyl)benzo[*c*]isoxazol-7-yl)benzoate **18**:

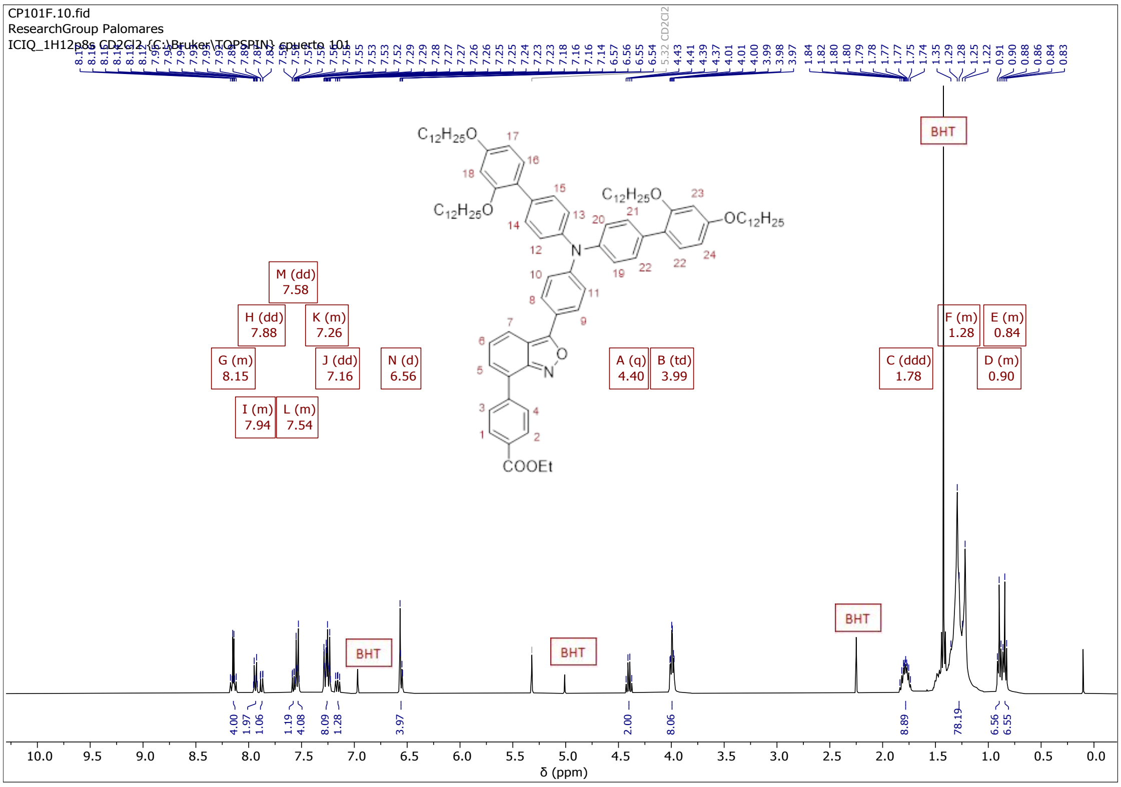

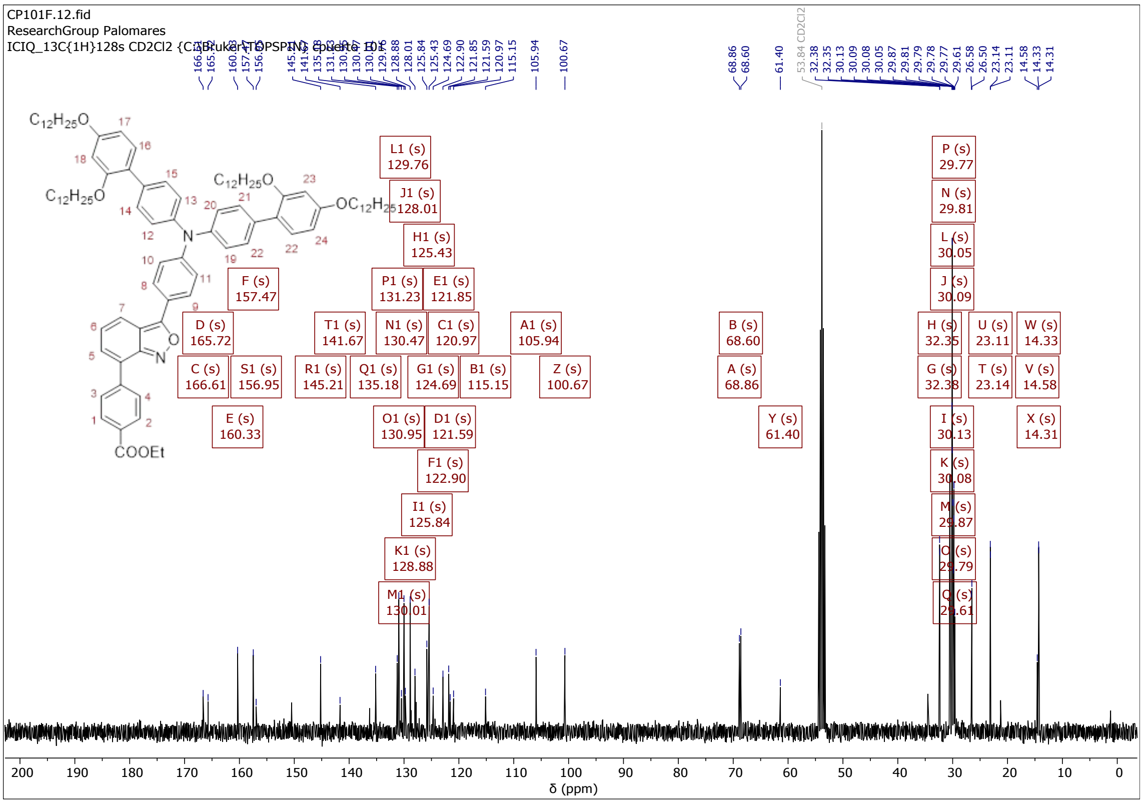

Figure S23. 1D- and 2D-NMR spectra of 4-(3-(4-(bis(2',4'-bis(dodecyloxy)-[1,1'-biphenyl]-4-yl)amino)phenyl)benzo[*c*]isoxazol-7-yl)benzoic acid **20**:

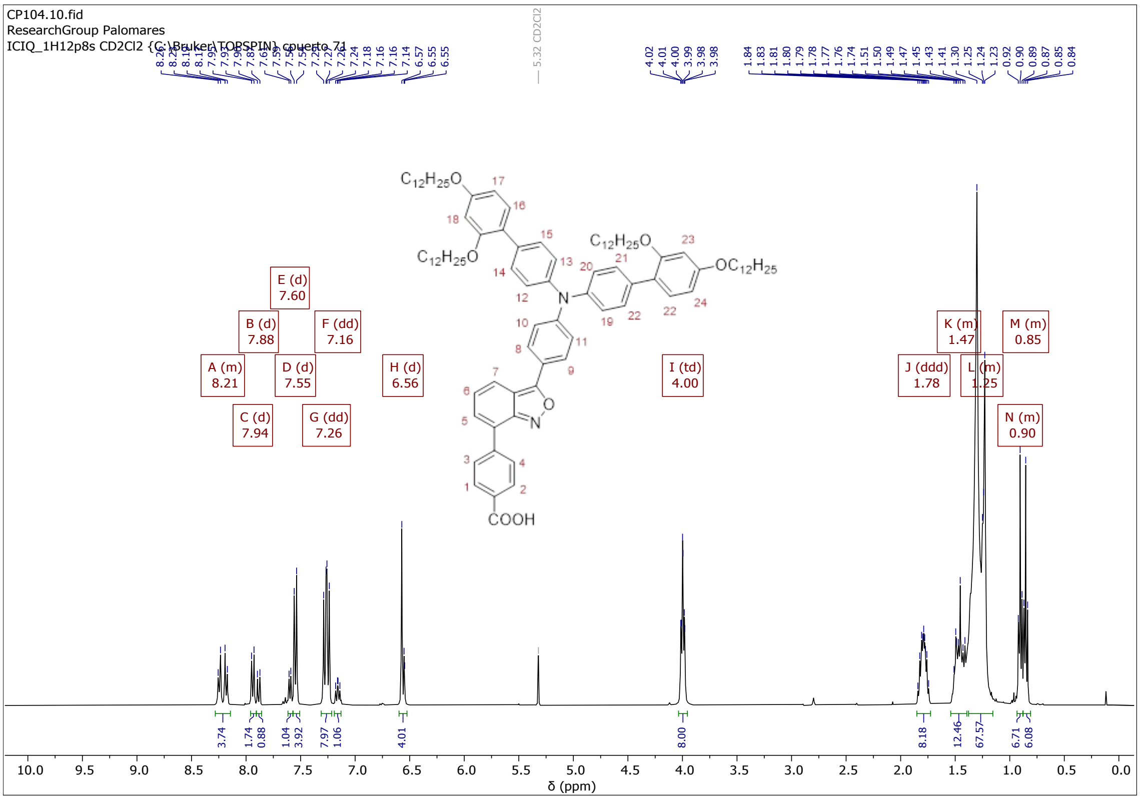

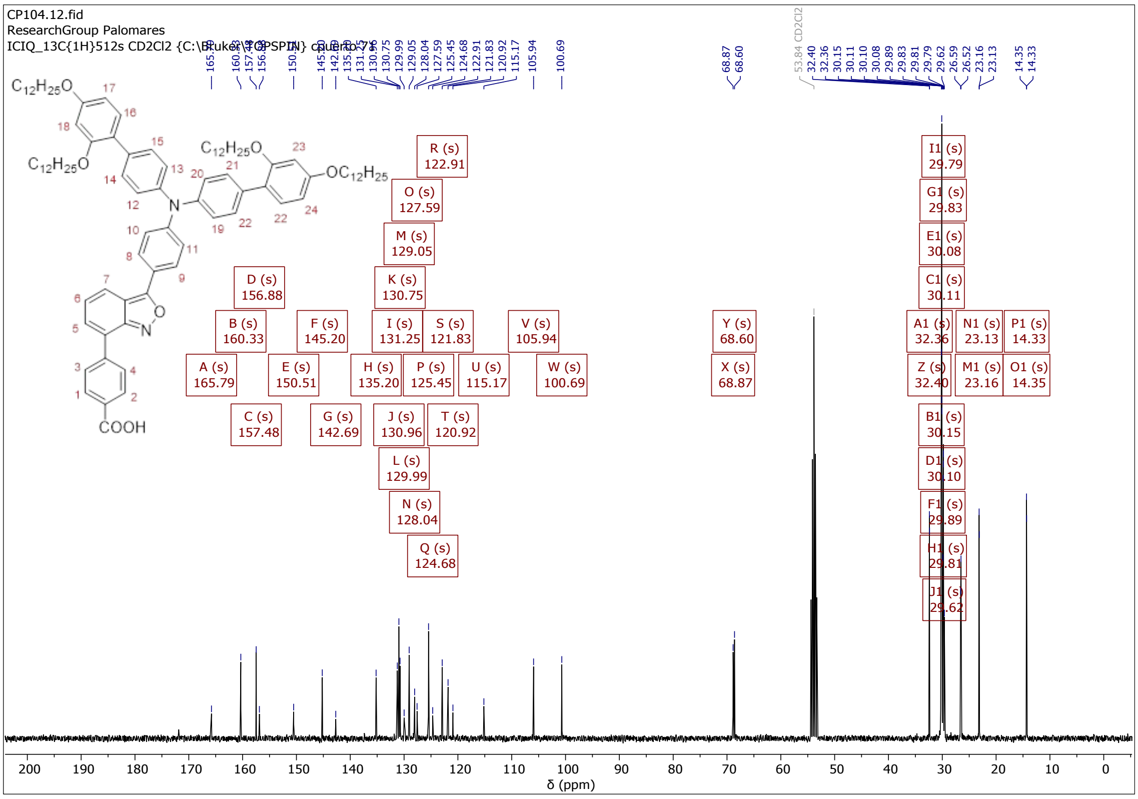

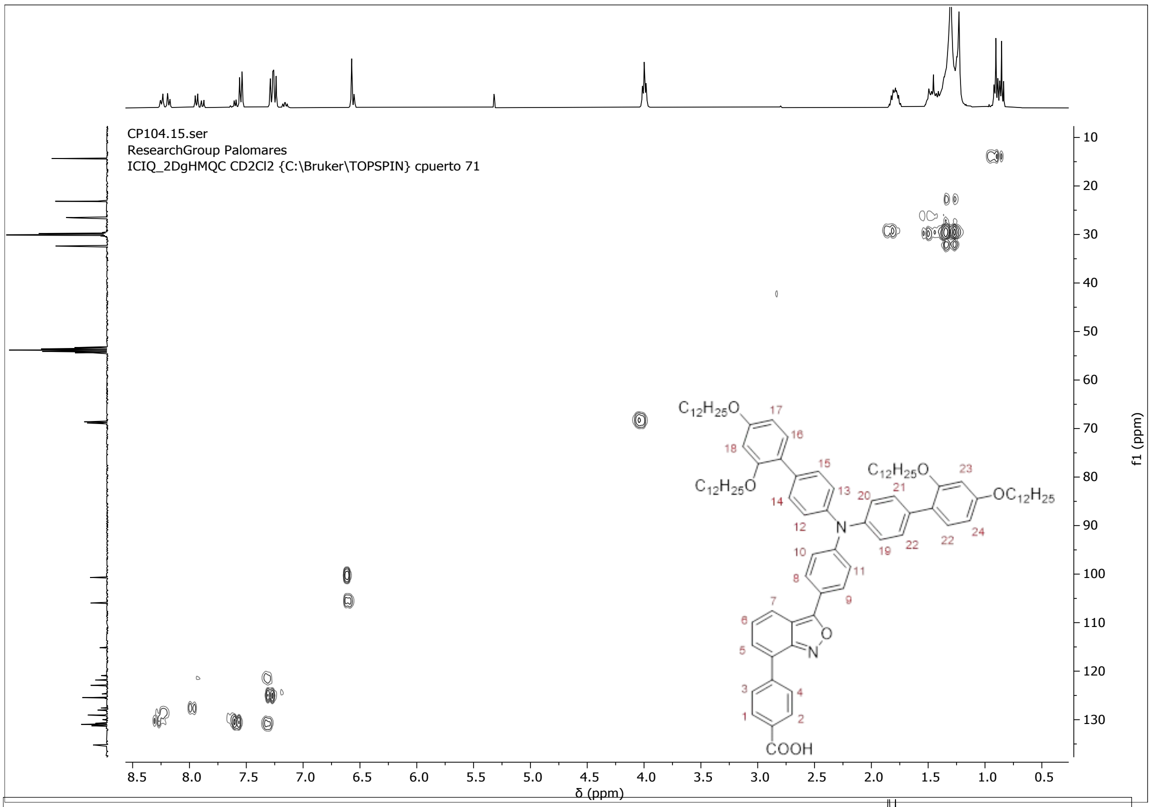
CP104.15.ser
ResearchGroup Palomares
ICIQ_2DgHMQC CD2Cl2 {C:\Bruker\TOPSPIN} cpuerto 71
δ (ppm)
f1 (ppm)
COOH

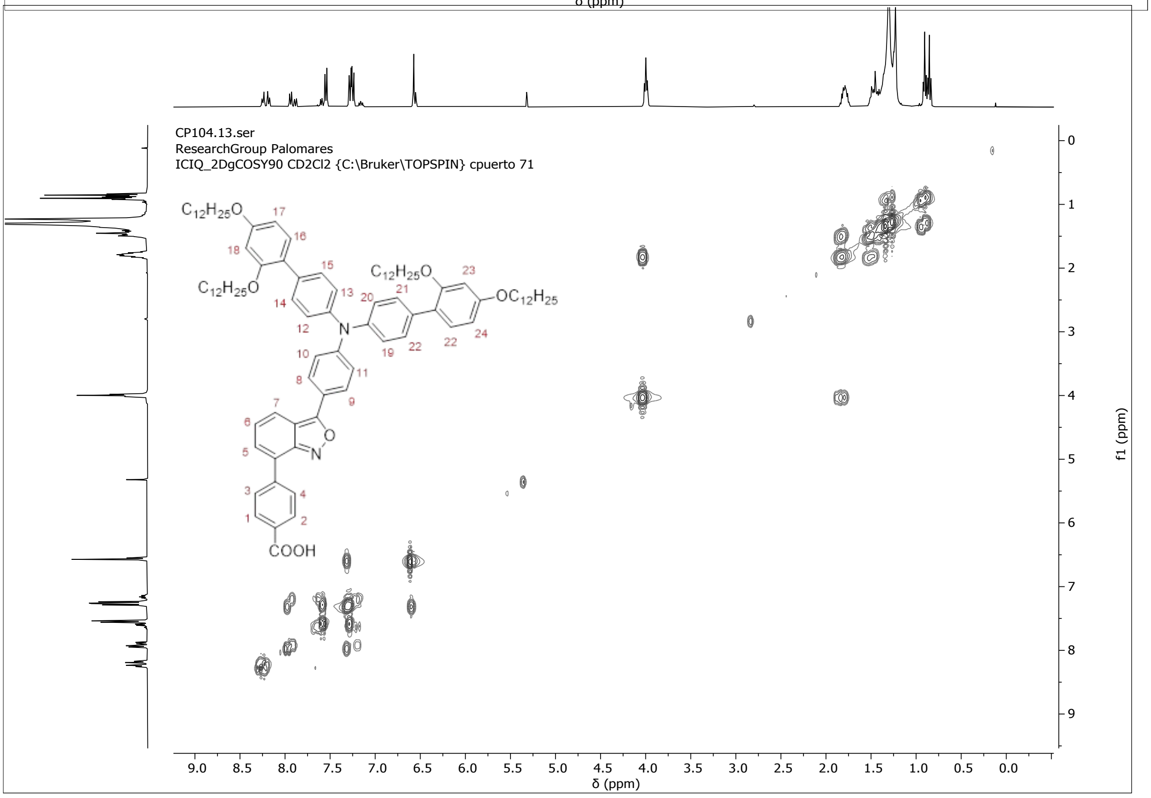
CP104.13.ser
ResearchGroup Palomares
ICIQ_2DgCOSY90 CD2Cl2 {C:\Bruker\TOPSPIN} cpuerto 71
δ (ppm)
f1 (ppm)
COOH

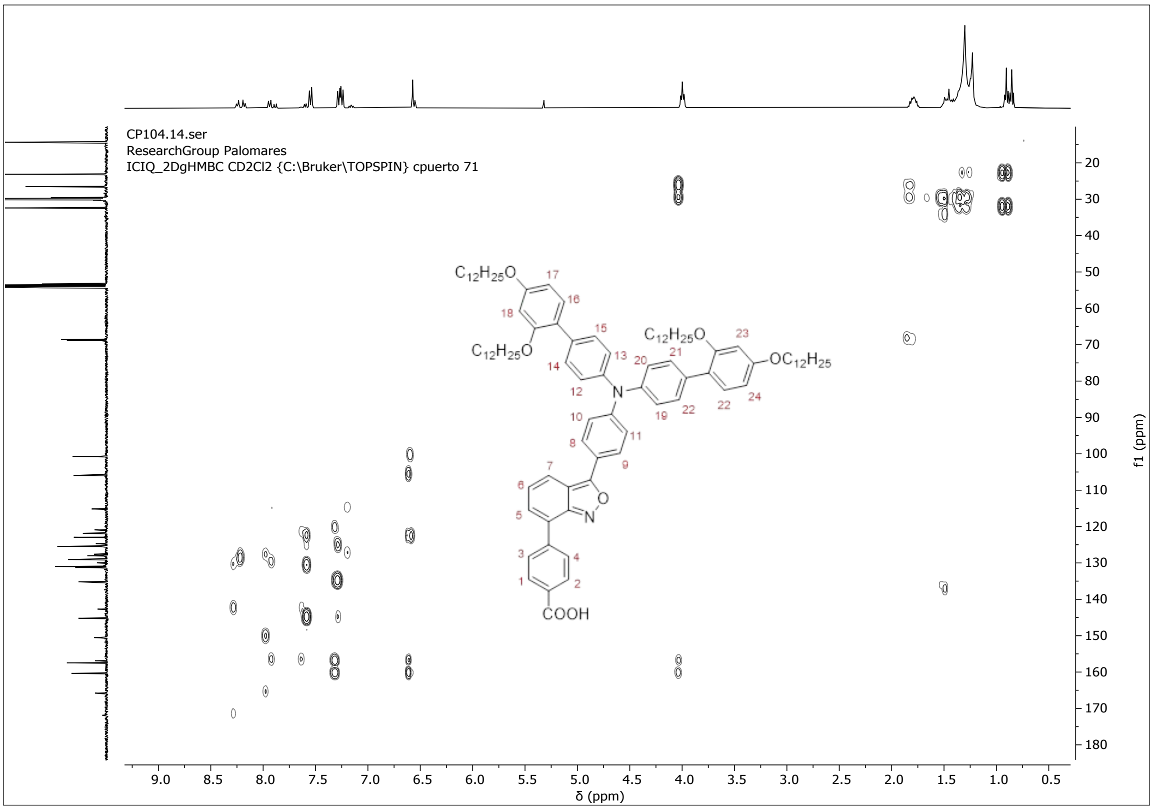
CP104.14.ser
ResearchGroup Palomares
ICIQ_2DgHMBC CD2Cl2 {C:\Bruker\TOPSPIN} cpuerto 71
δ (ppm)
f1 (ppm)
COOH
